# Development of an Autophagy-Based and Stemness-Correlated Prognostic Model for Hepatocellular Carcinoma Using Bulk and Single-Cell RNA-Sequencing

**DOI:** 10.3389/fcell.2021.743910

**Published:** 2021-11-08

**Authors:** Shengwei Shen, Rui Wang, Hua Qiu, Chong Li, Jinghan Wang, Junli Xue, Qinghe Tang

**Affiliations:** ^1^Department of Hepatobiliary and Pancreatic Surgery, Shanghai East Hospital, School of Medicine, Tongji University, Shanghai, China; ^2^The First Affiliated Hospital of Nanchang University, Nanchang, China; ^3^Department of Oncology, Shanghai East Hospital, School of Medicine, Tongji University, Shanghai, China

**Keywords:** autophagy, stemness, ATIC, BIRC5, prognosis

## Abstract

Accumulating evidence has proved that autophagy serves as a tumor promoter in formed malignancies, and the autophagy-related prognostic signatures have been constructed as clinical tools to predict prognosis in many high-mortality cancers. Autophagy-related genes have participated in the development and metastasis of hepatocellular carcinoma (HCC), but the understanding of their prognostic value is limited. Thereafter, LIMMA and survival analysis were conducted in both ICGC and TCGA databases and a total of 10 hub autophagy-related genes, namely, NPC1, CDKN2A, RPTOR, SPHK1, HGS, BIRC5, SPNS1, BAK1, ATIC, and MAPK3, were collected. Then, GO, KEGG, correlation, consensus, and PCA analyses were utilized to reveal their potential targeted role in HCC treatment. Single-cell RNA-seq of cancer stem cells also indicated that there was a positive correlation between these genes and stemness. In parallel, we applied univariate, LASSO, and multivariate regression analyses to study the autophagy-related genes and finally proposed that ATIC and BIRC5 were the valuable prognostic indicators of HCC. The signature based on ATIC and BIRC5 exhibited moderate power for predicting the survival of HCC in the ICGC cohort, and its efficacy was further validated in the TCGA cohort. Taken together, we suggested that 10 aforementioned hub genes are promising therapeutic targets of HCC and the ATIC/BIRC5 prognostic signature is a practical prognostic indicator for HCC patients.

## Introduction

As one of the most generally diagnosed and the predominant cause of cancer-associated death, hepatocellular carcinoma (HCC) still remains challenging to cure and demanding to better predict its prognosis (Siegel et al., 2017; Bray et al., 2018). Upon more and more diagnosis approaches, therapies of HCC also increase rapidly, including surgical resection, chemotherapy, hepatic artery embolization, and immunotherapy (Prieto et al., 2015). Nevertheless, the clinical outcome is unfavorable owing to the complexities and heterogeneity of intra- and inter-cancer cells. Therefore, it is imperative to develop individualized therapeutics. Moreover, discovery of applicable prognostic signature may aid in personalized treatment development.

Autophagy is the biological system that participates in the degradation of disable proteins, organelles, and cellular constituents, thereby managing cell homeostasis and energy balance (Liu B. et al., 2013). Previous studies have reported that dysregulation of autophagy is accountable for extensive pathological diseases, containing cancers (Ke et al., 2016), cardiovascular disorders (Zhang C. et al., 2017), diabetes (Fetterman et al., 2016), neurodegeneration (Xilouri et al., 2016), and aging (Redmann et al., 2016). Apart from taking important part in different cell disorders, autophagy also has a relatively bidirectional function in malignant cells. During the initiation of cancer, autophagy could resist tumor proliferation by eliminating aberrant cytosolic components like altered proteins and organelles. Thus, autophagy protects normal cells and tissues from DNA vulnerability and genomic alteration at the early stage of tumors (Jin and White, 2007). However, once the cancer has established and developed, autophagy, functioned as a cell death controller in mature cancer cells (Mathew et al., 2007; Bhutia et al., 2013), is capable of supporting tumor growth *via* preserving neoplasms from stressful conditions, involving necrosis and inflammation, hypoxia, and nutrient deficiency (Degenhardt et al., 2006; Lorin et al., 2013). Sui et al. (2013) demonstrated that autophagy is a potential target for anticancer therapy and the formation of autophagy relating to therapeutics can be identified as drug resistance and disease progression. Over the recent years, the autophagy-related gene signature has been constructed to predict prognosis in multiple tumors, including resected pancreatic cancer (Ko et al., 2013), glioma (Zhang H. et al., 2017), and breast tumor (Gu et al., 2016). However, the specific relationship between autophagy and HCC has not yet been fully elucidated by far.

In view of available and online extensive RNA-seq data, we sought to investigate whether the gene expression pattern of autophagy-related genes would be able to predict prognosis for HCC patients. In the current research, we firstly collected 232 autophagy-related genes from HADb (Human Autophagy Database^1^). Then, we used two well-known databases—ICGC (The International Cancer Genome Consortium^2^) and TCGA (The Cancer Genome Atlas^3^)—to extract the autophagy-associated mRNA expression for further study. Additionally, we analyzed various differentially expressed and autophagic genes for HCC patients. To highlight the significance of these potential hub genes, correlation, GO, KEGG, consensus, PCA, and survival analyses were all conducted to comprehensively illuminate the molecular properties and undeveloped roles of these genes in HCC. Furthermore, single-cell RNA-seq (scRNA-seq) data were also utilized to further understand their oncogenic roles. On the other hand, we particularly focused on the predictive value of autophagy-associated hub genes and assembled an accurate prognosis signature based on the ICGC dataset, and the power of the model was re-confirmed by the TCGA dataset. Finally, we surmised a nomogram to better estimate the overall survival for HCC patients. Overall, our data revealed that autophagy-related genes act essentially in liver cancer progression and are the potential prognostic indicators for HCC patients.

## Materials and Methods

### Data Processing

The International Cancer Genome Consortium (ICGC, see text footnote 2), established in 2007, solved many data governance, ethical, and logistical problems, thereby sharing the international community with inclusive genomic data for many cancer types. “ICGC-LIRI-JP,” one of the HCC RNA-seq data from RIKEN, Japan, was obtained from ICGC in the present study. Another set of sequence-based mRNA expression data of HCC was acquired from The Cancer Genome Atlas (TCGA, see text footnote 3). In our present work, ICGC was regarded as the discovery cohort (normal tissues = 202; tumor tissues = 243) while TCGA was set as the validation cohort (normal sample = 50; tumor sample = 374). Moreover, clinicopathological characteristics of 260 HCC patients from ICGC and 376 HCC patients from TCGA were also obtained for further investigation.

On the other hand, the scRNA-seq (GSE103866) of HCC cancer stem cells (CSC), sequenced by Smart-seq2 methods, was obtained from Gene Expression Omnibus (GEO^4^) (Zheng et al., 2018).

Human Autophagy Database (HADb, see text footnote 1), the first Human Autophagy-dedicated Database, delivers a comprehensive and latest repository of human genes and proteins contained directly or indirectly in autophagy as depicted in literature (Moussay et al., 2011). Therefore, 232 autophagy-related genes were collected from HADb. Based on these 232 genes, we extracted the corresponding gene expression matrix from the aforementioned ICGC and TCGA data for further analysis.

### Identification of Autophagy-Related Differentially Expressed Genes in Hepatocellular Carcinoma

**FIGURE 1:**
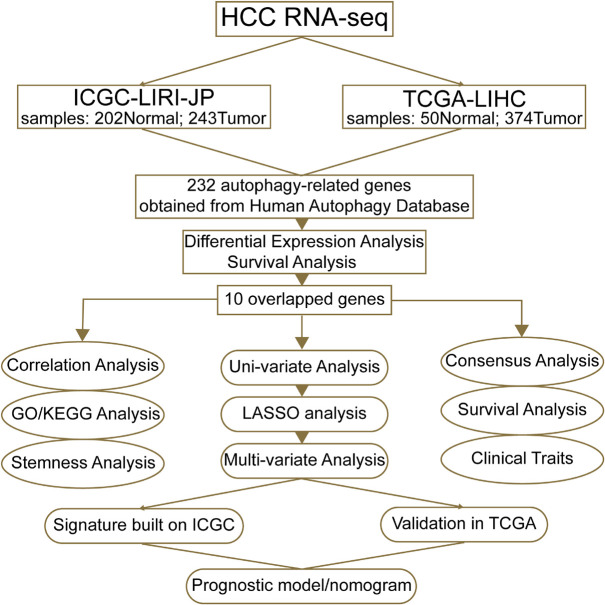
A workflow of our current work. “ICGC-LIRI-JP” denotes that the liver cancer data of the ICGC database were obtained from RIKEN, Japan. “TCGA-LIHC” means that data of liver hepatocellular carcinoma were from TCGA database.

The autophagy-related gene expression datasets were employed to identify the differentially expressed genes. The specific flowchart of this research is shown in Figure 1. Firstly, we used LIMMA analysis to get the DEGs by comparing tumor specimens with non-tumor specimens in ICGC and TCGA, respectively. According to the filtering criteria (adjusted *p* < 0.05 and fold change > 2), 25 DEGs in ICGC and 54 DEGs in TCGA were identified. The detailed analytic information of the autophagy-related genes is presented in Supplementary Tables 1, 2.

### Discovery of the Prognosis-Related and Autophagy-Related Differentially Expressed Genes in Hepatocellular Carcinoma

Among 25 autophagy-related DEGs in ICGC and 54 autophagy-related DEGs in TCGA, Kaplan–Meier survival analysis was performed to figure out the potential prognostic DEGs. *p*-value < 0.05 was regarded as significance. To obtain more precise results, a Venn plot was utilized to identify the shared prognostic DEGs in both ICGC and TCGA. As a result, 10 shared hub genes were obtained for deeper study.

### Correlation and Functional Analyses of 10 Hub Genes

To further confirm the underlying function of potential targets, the data were analyzed by functional enrichment. In the present work, we employed the R ClusterProfiler package (R version: 3.12.0) to analyze the Gene Ontology (GO) and Kyoto Encyclopedia of Genes and Genomes (KEGG) function of 10 hub genes. Furthermore, adjusted *p* < 0.05 was used to filter the functional results.

### Single-Cell RNA-Sequencing Data Analysis

Smart-seq2 data of different types of HCC cancer stem cells were downloaded, including three CSC samples: Huh-1 cell line, Huh-7 cell line, and patient-derived CSCs. After aggregation of the three samples, R package Seurat (v4.0.4) was used for cell filtration, PCA, top-2,000 highly variable gene finding, clustering analysis, and dimensional reduction. Cells with a percentage of mitochondrial sequencing count > 30%, “min.cells < 3,” and “min.features < 300” were excluded. The Seurat functions, involving DotPlot, Vlnplot, FeatureScatter, and FeaturePlot, were employed to visualize the autophagy-related genes, EPCAM, CD24, and CD133 expressions, respectively. To remain the heterogeneity as more as possible, the data transformation was not further conducted (Zheng et al., 2018). Finally, the analyzed data in the present work contained 130 single or pooled cells, which included 55 HuH-1 cells, 63 HuH-7 cells, and 12 patient HCC cells.

### Consensus Cluster Analysis and PCA Analysis

To investigate the function of 10 hub genes in HCC, we clustered the HCC into various subgroups with an R package, ‘‘ConsensusClusterPlus’’ (Version 1.48.0^5^). Then, PCA in R (R version: 3.5.1) was conducted to investigate the gene expression patterns in the appropriate stratified subgroups. Finally, survival analysis and clinical information were used to validate the importance of the cluster results of 10 hub genes in the ICGC and TCGA databases.

### Construction and Validation of a Prognostic Model

To determine the prognostic value of autophagy-related genes, we performed univariate Cox regression analysis of 10 hub genes in ICGC, the discovery cohort in our study. Then, those with *p*-value < 0.01 were imported to run the least absolute shrinkage and selection operator (LASSO) Cox regression algorithm, thereby making the least and best risk factors to construct a prognostic model. By performing the multivariate Cox regression analysis, we further confirm the significant role of ATIC and BIRC5 in HCC. Moreover, survival curves, ROC curves, and risk score distributions of HCC patients were plotted in both ICGC and TCGA data for validation of the signature.

### Development of an Applicable Nomogram for Individualized Treatment

After confirming the prognostic value of an ATIC/BIRC5 signature, the nomogram algorithm (see text footnote 5) in R software was conducted for HCC patients based on the combination of this prognostic signature and relevant clinical traits.

## Results

### Identification of Differentially Expressed Genes Related to Autophagy in Hepatocellular Carcinoma

**FIGURE 2:**
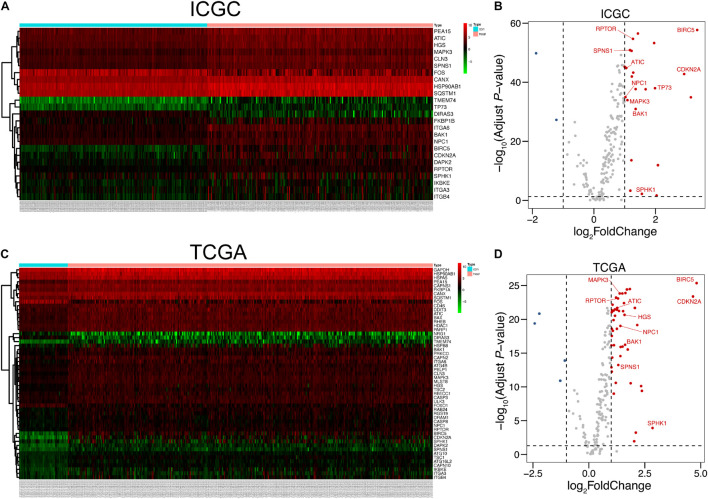
Autophagy-related DEGs of HCC in ICGC and TCGA. **(A,C)** Heat maps of DEGs in ICGC and TCGA. The number of normal tissues was 202, and the number of tumor tissues was 243, all retrieved from ICGC. As for TCGA, the number of normal samples was 50 and that of tumor samples was 374. **(B,D)** Volcano plots of DEGs in the ICGC and TCGA databases. Ten hub genes studied in our research were labeled in the corresponding position. The detailed analytic information of the autophagy-related genes is presented in Supplementary Tables 1, 2.

The overall procedure of our present work is shown in Figure 1. By virtue of the vital role of autophagy in malignancies, we firstly found out the expression data of HCC in ICGC and TCGA based on 232 autophagy-related genes collected from HADb (see text footnote 1). After data normalization, LIMMA analysis in R was performed to explore DEGs in HCC patients in ICGC and TCGA, respectively. We figured out a total of 25 autophagic DEGs in ICGC, including two downregulated genes and 23 upregulated genes (Figures 2A,B), and a total of 54 DEGs in TCGA (Figures 2C,D), containing four downregulated genes and 50 upregulated genes (fold change > 2, adjusted *p* < 0.05).

### Survival Analysis of Autophagic Differentially Expressed Genes in ICGC and TCGA

**FIGURE 3:**
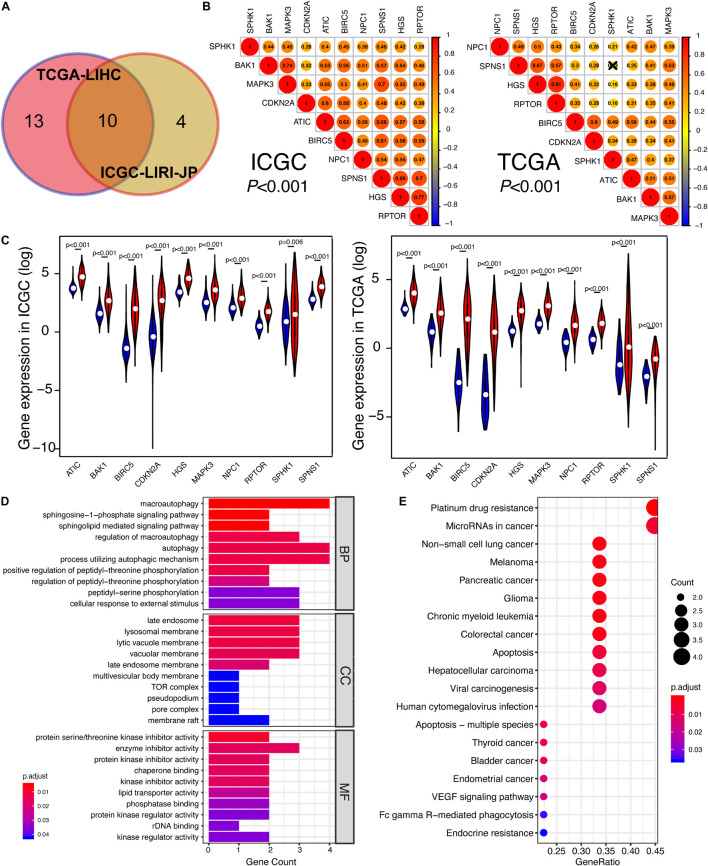
The identification of prognosis-related DEGs in ICGC and TCGA. **(A)** Venn plot of prognosis-related DEGs between ICGC and TCGA. **(B)** The correlation among 10 overlapped prognosis-related DEGs in ICGC and TCGA. The closer the correlation between two genes, the bigger the circle size is. **(C)** Gene expression of 10 hub genes in ICGC and TCGA. The blue color represents the gene expression in normal specimens, while the red color represents the gene expression in tumor specimens. **(D)** The GO analysis of 10 hub genes, including these following terms: biological process (BP), molecular function (MF), and cellular component (CC). **(E)** The KEGG analysis of 10 hub genes. “Adjust *p* < 0.05” was the criterion.

**FIGURE 4:**
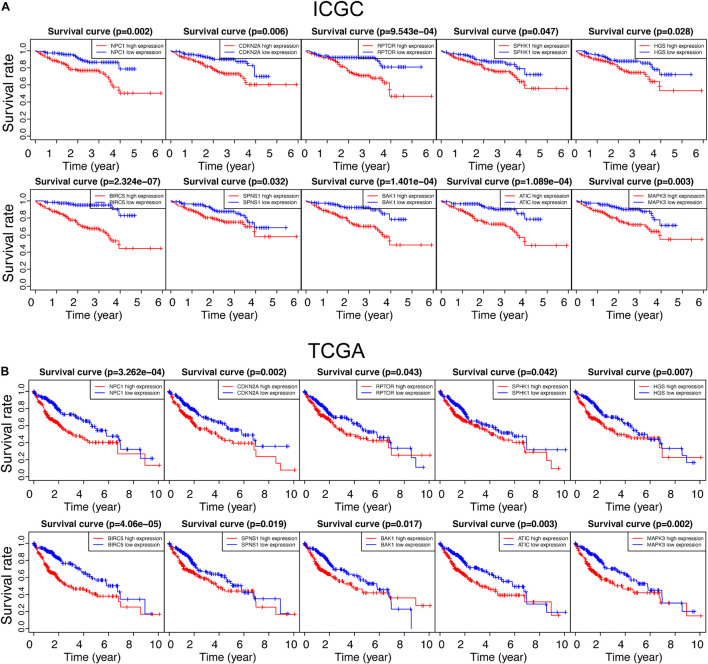
Survival analysis of all autophagy-related DEGs in the ICGC and TCGA databases. “*p* < 0.05” was considered significant, and only 10 overlapped genes in ICGC and TCGA are presented in panels **(A,B)**, respectively.

To investigate the relationship between autophagic DEGs and prognosis of HCC patients, we adopted the overall survival (OS) analysis in 25 autophagic DEGs in ICGC and 54 autophagic DEGs in TCGA. As a result, 14 out of 25 DEGs and 23 out of 54 DEGs were considered significant in ICGC and TCGA, respectively (*p*-value < 0.05). After identifying the intersected genes in both ICGC and TCGA, only 10 hub genes were left for further analysis (Figure 3A). As shown in Figures 4A,B, the OS analysis of 10 hub genes, including NPC1, CDKN2A, RPTOR, SPHK1, HGS, BIRC5, SPNS1, BAK1, ATIC, and MAPK3, is presented. Moreover, we found that the high expression of these 10 hub genes predicts poor diagnosis for HCC patients in the ICGC and TCGA datasets, consistent with the tumor-promoting role of autophagy-related genes in the development of established malignancies (White, 2012; Amaravadi et al., 2016).

### The Correlation Among 10 Hub Targets Were Close and Functional Analysis Revealed They Participated in Various Cancers

With the purpose of better understanding the expression pattern and underlying function of 10 hub genes, correlation analysis was conducted. As shown in Figure 3B, with strict criteria (*p*-value < 0.001), 10 hub genes still exhibited strong relativity with each other in both ICGC and TCGA. On the other hand, we found out that the expressions of these 10 hub genes in HCC specimens were all significantly higher than those of non-tumor specimens in both ICGC and TCGA (Figure 3C), indicating the potential carcinogenesis function of the 10 autophagy-related genes in HCC. Based on GO annotations, Figure 3D shows that the MF, CC, and BP of 10 hub genes were all pointed to the specific activity and process of autophagy, confirming the close relationship between 10 hub genes and autophagy. Regarding KEGG analysis, various KEGG pathways were enriched like “Non-small cell lung cancer,” “Melanoma,” “Pancreatic cancer,” “Glioma,” “Colorectal cancer,” “Apoptosis,” “Hepatocellular carcinoma,” and “Viral carcinogenesis” (Figure 3E). Therefore, we draw a conclusion that 10 hub genes took an important position in several cancers involving HCC and might be promising targets for HCC therapies.

### Most 10 Autophagy- and Prognosis-Related Genes Were Positively Correlated With EPCAM, CD24, and PROM1

**FIGURE 5:**
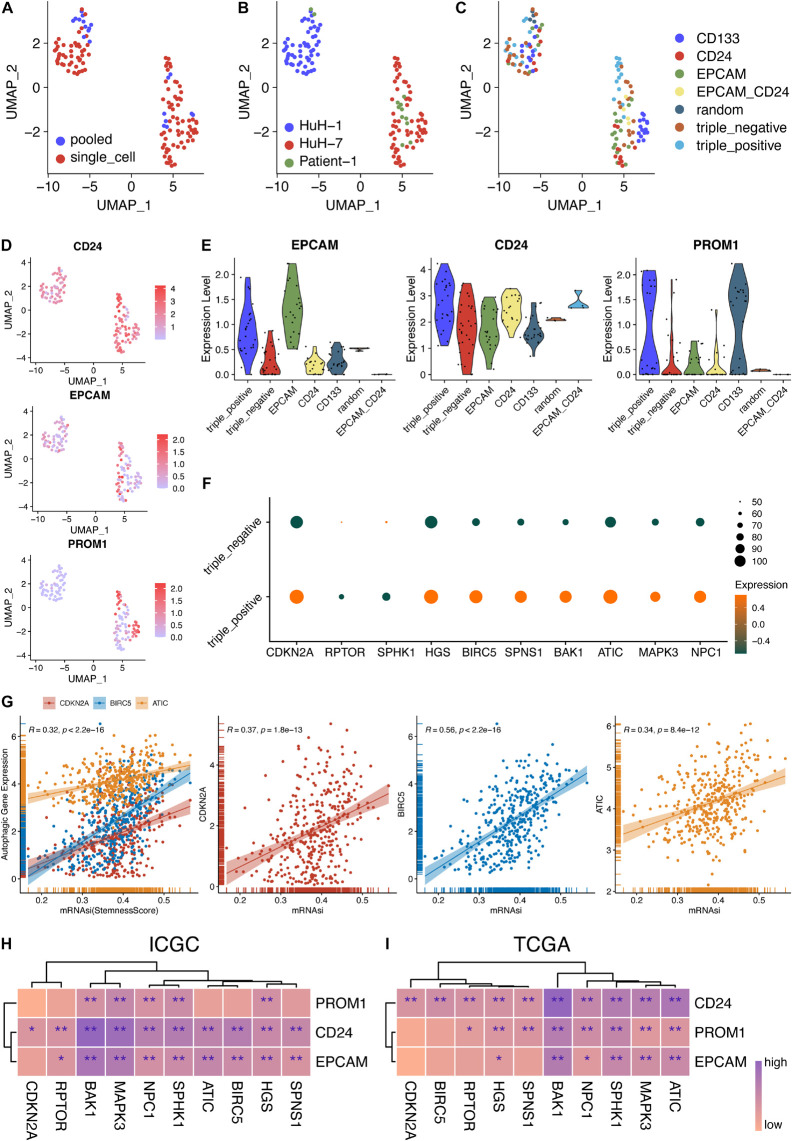
Stemness analysis of 10 hub genes in bulk and single-cell RNA-seq data. **(A–C)** UMAP plot of 130 single cells using different classifications. **(D)** Expression levels of three CSC markers in single-cell and pooled-cell samples. **(E)** The expression levels of the three CSC markers were consistent with the FACS-sorting results. **(F)** Expression levels of 10 autophagy genes in triple-negative and triple-positive samples. **(G)** Correlation analysis between autophagy genes and stemness scores in TCGA-LIHC patients. **(H)** Correlation analysis between 10 autophagy genes and three CSC markers in the ICGC database. **(I)** Correlation analysis between 10 autophagy genes and three CSC markers in the TCGA database. *P < 0.05 and **P < 0.01.

Now that the KEGG analyses of these 10 genes were enriched in various cancers, it triggered us to consider their oncogenic roles in HCC more than only regulating autophagy. CSCs are specific cell clusters that are believed to take responsibility for cancer recurrence, metastasis, and chemoresistance, thereby resulting in poorer prognosis. Moreover, studies suggest that CSC maintenance and differentiation also depend upon autophagy (Suetsugu et al., 2006; Camuzard et al., 2020; Yao et al., 2020; Wang et al., 2021). Therefore, we collected a dataset (GSE103866) which contains various kinds of hepatic CSCs from two HCC cell lines and patient-derived CSCs. After normalization, 130 single or pooled cells, involving 100 single-cell and 20 pooled cell samples, were distributed mainly by their sources and HuH7 cells were more similar to patient cells rather than HuH1 cells (Figure 5B and Supplementary Figures 1A,D). Meanwhile, 20 pooled cell samples were composed of eight 10-pooled-cell samples and 12 100-pooled-cell samples, which mean that eight samples had 10 cells per pooled sample while 12 samples had 100 cells per pooled sample (Supplementary Figures 2B,C).

Additionally, the sequencing depth of pooled-cell samples was higher than that of single-cell samples (Figure 5A and Supplementary Figures 1B,E). The detected gene numbers and the sequencing depth were positively correlated (Pearson’s *r* = 0.87) (Supplementary Figures 1C,F). Then, three markers of CSCs were all expressed in these three sources, respectively (Figures 5B–D). Furthermore, the expression levels of EPCAM, CD24, and PROM1 were consistent with their sorting from EPCAM, CD24, and CD133 using fluorescence-activated cell sorting (FACS), respectively (Figure 5E). The variance analysis showed the top 5,000 variably expressed genes across all cell samples (Supplementary Figure 2D). Furthermore, principal component analysis (PCA), the linear dimensionality reduction method, was conducted and the significantly components were screened (Supplementary Figures 2E,F). The top 15 components were calculated and selected for subsequent analysis. Then, the Uniform Manifold Approximation and Projection (UMAP) algorithm, a non-linear dimensionality reduction method, was adopted for further precisely clustering the potential cell populations (Supplementary Figure 2A). Interestingly, only two clusters were obtained and they were not grouped by CSC markers. More precisely, the clusters were aggregated according to their sources, indicating the distinct heterogeneity of HuH-1 and HuH-7 cell lines (Supplementary Figures 2A,C).

Provided that the scRNA-seq data of CSCs were normalized and reliable, we tended to investigate the relationship between these 10 autophagy-related genes and cancer cell stemness. As shown in Figure 5F, we found that most autophagic genes were highly expressed in triple-positive CSCs than in triple-negative CSCs, indicating that the high expression of the autophagic genes might enhance the stemness of cancer cells and thereafter result in poorer prognosis. To validate the scRNA-seq results, we further explored the correlation between 10 hub genes and three CSC markers. Concordantly, almost 10 hub genes were significantly and positively correlated with EPCAM, CD24, and PROM1 (CD133), respectively, whether in ICGC patients (Figure 5H and Supplementary Figure 3) or TCGA patients (Figure 5I and Supplementary Figure 4). Malta et al. (2018) reported the well-calculated stemness scores for TCGA HCC patients. Therefore, we also analyzed the correlation between stemness scores and the 10 hub genes in our work. In line with aforementioned results, three of 10 hub genes, ATIC, BIRC5, and CDKN2A, also had a significant and positive correlation with stemness scores of HCC patients (Figure 5G) while others had a positive correlation but not significant (data not shown). These results indicated that 10 autophagy-related genes might also affect the stemness of HCC.

### Consensus Analysis and PCA Analysis Disclosed That 10 Hub Genes Could Be an Applicable Criterion to Stratify Hepatocellular Carcinoma Patients Into Different Subgroups

**FIGURE 6:**
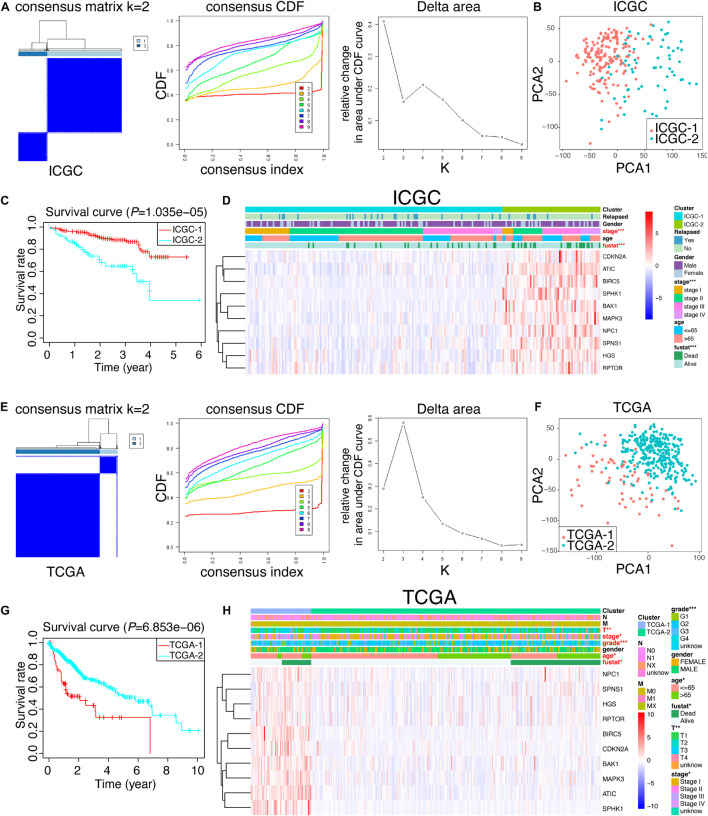
Consensus cluster analysis and PCA analysis. **(A,E)** Cumulative distribution function (CDF) and relative change in the area under the CDF curve of the consensus cluster for *k* = 2–10 in ICGC and TCGA; *k* = 2 was presented and selected for further analysis. **(B,F)** PCA analysis of the RNA-seq profile of HCC patients in ICGC and TCGA. **(C,G)** Kaplan–Meier survival curves for HCC patients in ICGC and TCGA. **(D,H)** Heat map and clinicopathologic traits of the two clusters (cluster 1 and cluster 2) defined by the 10-hub-gene consensus expressions of ICGC and TCGA, respectively.

Given the potential crucial role of these 10 hub genes, we tried to figure out whether they could be utilized to identify the molecular subtypes of HCC patients. Based upon the expression similarity of 10 hub genes (Figures 6A,E), we selected “*k* = 2” as a criterion, which was generally able to cluster HCC patients into two subgroups appropriately in ICGC and TCGA, respectively. Moreover, other k results were not shown because the consistency between the two datasets was not superior to “*k* = 2” (data not shown). Moreover, we performed PCA analysis to better visualize the subgroups, containing ICGC-1, ICGC-2, TCGA-1, and TCGA-2. As revealed in Figures 6B,F, we demonstrated that not only in ICGC but also in TCGA, the whole gene expression patterns of HCC patients could separate into two clusters properly based on only 10 hub autophagic DEGs. Furthermore, ICGC-1 and ICGC-2, based upon this 10-hub-gene classification, had a significantly different prognosis of HCC patients (Figure 6C). Likewise, we could also draw a similar conclusion that TCGA-1 has a different prognosis of HCC patients compared with TCGA-2 (Figure 6G). Thus, we further compared the clinicopathological traits of these two subgroups in ICGC and TCGA. The ICGC-2 subgroup was significantly correlated with higher 10-hub-gene expression, higher stage (*p*-value < 0.001), and worse survival status (*p*-value < 0.001) (Figure 6D). On the other hand, TCGA-1 was not only characterized by higher 10-hub-gene expression, higher stage (*p*-value < 0.05), and worse survival status, but closely relative to higher tumor stage (*p*-value < 0.01), higher grade (*p*-value < 0.001), and older age (*p*-value < 0.05) (Figure 6H).

### A Prognostic Signature Was Built in the ICGC Cohort and Validated in the TCGA Cohort

**FIGURE 7:**
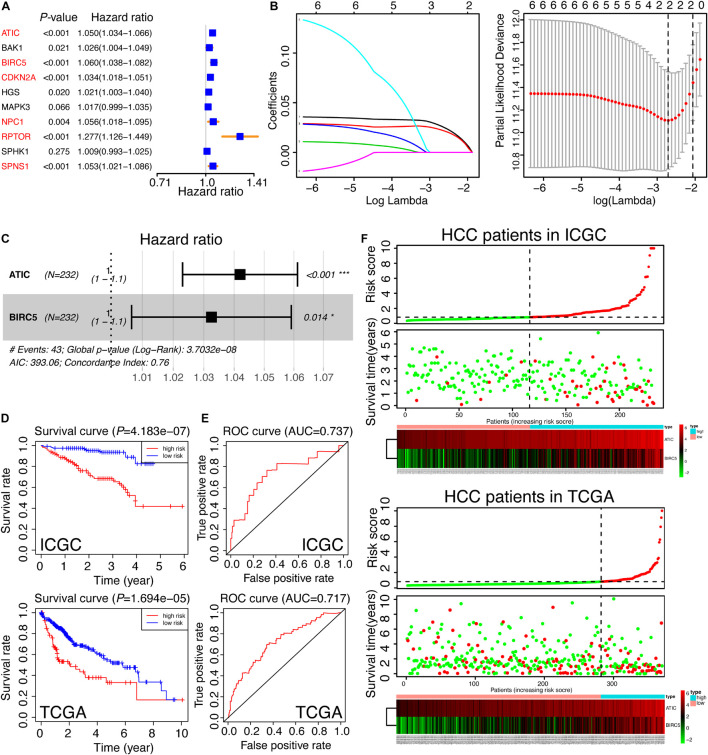
Construction and validation of the autophagy-related gene signature. **(A)** The univariate analysis of 10 hub genes for HCC patients in ICGC, which was classified as a training set in our study. The genes in red color were recognized as significance (*p* < 0.01). **(B)** The selection of risk factors by LASSO analysis. **(C)** Multivariate Cox analysis of ATIC and BIRC5 in ICGC and the prognostic signature were constructed. The formula was as follows: RiskScore = 0.04 × ATIC + 0.03 × BIRC5. The Concordance Index was 0.76. **(D)** The survival curves of HCC patients based on the RiskScore signature in the training set (ICGC) and the testing set (TCGA). **(E)** The ROC curves calculated by the RiskScore signature in ICGC and TCGA. **(F)** The thermal maps and distributions of the two gene expression profiles in the high- and low-risk subgroups in the training set and the testing set, respectively. The red circle represents “Dead” while the green circle represents “Alive.” The green curve denotes “low-risk,” and the red curve means “high-risk.” The risk scores of HCC patients in TCGA were calculated by the RiskScore signature and divided into two subgroups according to the medium score of the training set.

Considering the strong relation between 10 hug genes and prognosis of HCC, we next sought to investigate the prognostic value of these 10 autophagic DEGs in HCC. We performed a univariate analysis on the expression levels in ICGC, the discovery cohort (Figure 7A). The results indicated that six out of 10 candidate genes are significantly correlated with clinical outcomes (*p-*value < 0.01). The six genes, comprising ATIC, BIRC5, CDKN2A, NPC1, RPTOR, and SPNS1, were all oncogenes with hazard ratio > 1. To better predict the clinical outcomes of HCC, we utilized the least absolute shrinkage and selection operator algorithm (LASSO) to the six prognosis-related genes in the ICGC cohort (Figure 7B). As a result, only two hub genes were chosen to construct the prognostic model according to the minimum criteria. Moreover, the multivariate Cox analysis was applied to further validate the results and obtain the associated coefficients. Furthermore, the detailed formula was as follows: RiskScore = 0.04 × ATIC + 0.03 × BIRC5 (Figure 7C). According to this risk score model, we calculated the risk score for both the discovery cohort (ICGC) and the validation cohort (TCGA). To explore the predictive value of the autophagy-associated signature, we divided HCC patients in the ICGC and TCGA cohorts into low- and high-risk subgroups developed on the median risk score of ICGC, the discovery cohort. Also, the OS curves and ROC curves were plotted. As shown in Figure 7D, the high-risk group predicted worse prognosis for HCC patients in ICGC and TCGA (*p*-value < 0.01). Then, the area under the ROC curve (AUC) was 0.737 in the discovery cohort and 0.717 in the validation cohort, indicating the preferable prognostic value of this signature (Figure 7E). Finally, the distribution of risk scores in individual HCC patients and related survival status are exhibited in Figure 7F. We could conclude that the higher the risk score, the worse the survival status was.

### A Novel Nomogram Was Established for Hepatocellular Carcinoma Patients

**FIGURE 8:**
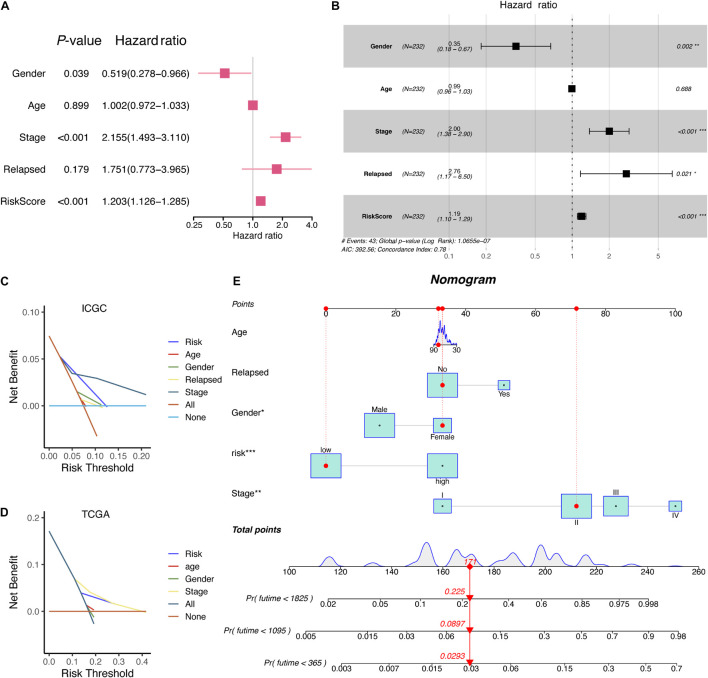
The corresponding nomogram developed from the RiskScore signature and clinical characteristics of HCC patients in ICGC. **(A)** Univariate Cox analysis of clinical information. **(B)** Multivariate Cox analysis of clinical information. **(C)** DCA results of RiskScore signature and clinical characteristics in the ICGC database. **(D)** DCA results of RiskScore signature and clinical characteristics in the TCGA database. **(E)** A nomogram based on RiskScore signature and related clinical information.

Given that a comprehensive analysis of HCC patients could better predict the clinical outcomes, we further adopted uni- and multivariate Cox regression algorithms to analyze the correlation between the clinical information and HCC patients. It presented that gender, stage, relapse, and RiskScore were the essential risk factors of HCC patients (*p*-value < 0.05) (Figures 8A,B). Moreover, the decision curve analysis (DCA) in both ICGC and TCGA showed that the RiskScore model might be comparable with pathologic stage (Figures 8C,D). Aiming at providing clinicians with a quantitative method to predict the OS of HCC patients, we made a nomogram that combined the RiskScore signature with different clinicopathologic risk factors. As shown in Figure 8E, the novel nomogram was able to evaluate the 1-, 2-, and 3-year survival probabilities. Moreover, it also indicated that the risk score was the most vital factor among the variables involved.

## Discussion

Hepatocellular carcinoma, the fourth major cause of cancer mortality worldwide is responsible for 80–90% cases of liver-related malignancies (Bray et al., 2018). By virtue of asymptomatic characteristics at the early stage of HCC, most HCC patients are diagnosed at advanced stages, resulting in poor prognosis. Therefore, along with the progress in surgical treatment and related adjuvant remedies, the efficiently diagnostic approach for identifying heterogeneous HCC patients is another invaluable way to improve the HCC prognosis.

RNA-seq biological technologies are being commonly applied to study HCC recurrence and metastasis by identifying altered genes (Roessler et al., 2010; Yuan et al., 2017). However, most predictive models were based on unexplored genes, which make it difficult for clinicians to explain the specific function of the members in prognostic signatures and might fail to figure out the potential targets obtained from signature for personalized therapy development. Therefore, developing a function-specific signature may better explain the innate function of related genes and further confirm the potential therapeutic molecules. In our present study, we focused on analyzing, constructing, and validating the autophagy-related and stemness-correlated gene signature and nomogram in HCC, thereby thoroughly understanding the relation between autophagy and HCC.

Previous studies have reported that autophagy-related signatures could exert effectively in many tumors. For example, Luan et al. (2019) demonstrated that a 10-autophagy-related lncRNA signature has prognostic potential for glioma based on CGGA (the Chinese Glioma Genome Atlas microarray) and TCGA. Likewise, Zhang H. et al. (2017) suggested that glioma patients from the CGGA, GSE4412, and TCGA datasets can be separated into high- and low-risk subclasses based upon levels of MAPK8IP1 and SH3GLB1 expression. Gu et al. (2016) showed that a set of eight autophagy genes (BCL2, BIRC5, EIF4EBP1, ERO1L, FOS, GAPDH, ITPR1, and VEGFA) were responsible for overall survival in breast cancer according to TCGA, GSE3494, and GSE7390. Another study reported that an eight autophagy-related gene signature serves as an independent prognostic marker for serous ovarian cancer, relying on TCGA data (An et al., 2018). Consistent with our study, a recent study from Lin et al. (2018) has revealed that three autophagy-related genes (BIRC5, FOXO1, and SQSTM1) were closely associated with the clinical outcomes of HCC, which was discovered in the TCGA dataset and validated in GSE10143, GSE10186, and GSE17856. However, our study focused on the integrative analysis of ICGC and TCGA datasets. The autophagy-related DEG analysis and the corresponding survival analysis of all potentials were conducted in ICGC, and most were successfully validated in TCGA. scRNA-seq of HCC CSCs was also adopted to confirm the positive correlation between autophagy and stemness in HCC. Moreover, besides the development of an autophagy-related signature and nomogram, we also thoroughly revealed the predictive model of autophagy genes for HCC built in ICGC and validated in TCGA datasets, providing the promising autophagy-related targets for HCC patients.

In our study, we first obtained 10 intersected autophagy DEGs in the ICGC and TCGA datasets. Serving as autophagy-related genes, these 10 hub DEGs were all considered upregulated in tumor tissues and to worsen the clinical outcomes of HCC patients. Subsequent GO and KEGG enrichment analyses indicated that the enriched terms were related to autophagy and various cancers, including HCC, thereby confirming the tumor-promoting role of these 10 dysregulated autophagy-associated targets. CSCs might be a common factor among most malignancies, and studies suggest that CACs also have an autophagic phenotype; scRNA-seq of HCC CSCs was further analyzed. Results highlighted the positive correlation among 10 hub genes and three CSC markers, EPCAM, CD24, and PROM1, respectively. Furthermore, univariate, LASSO, and multivariate Cox regression analyses enable us to select two major risk factors (ATIC and BIRC5) for building signature and nomogram. ATIC, a 64-kDa bifunctional enzyme, contains AICAR transformylase (AICART) and IMP cyclohydrolase, the final two activities in *de novo* purine biosynthesis (Greasley et al., 2001; Vergis et al., 2001). The team from Li et al. (2017) demonstrated that ATIC can support HCC cell proliferation and propagation *via* modulating the AMPK-mTOR-S6 K1-S6 pathway, coinciding with the autophagic role in the HADb database and tumor promoter role in our study. On the other hand, BIRC5, a part of the anti-apoptosis family, is associated with the specific formation process of autophagy and the cell survival of HCC (Chang et al., 2014). Moreover, BIRC5 can suppress apoptosis (Zhang et al., 2014), promote cell growth (Sun et al., 2013), induce angiogenesis (Fernandez et al., 2014), and even cause radio- and chemotherapy resistance (Liu W. et al., 2013). Based on ATIC and BIRC5, the built RiskScore signature in our study was capable of stratifying HCC patients in ICGC and TCGA into two prognosis-related subgroups. The RiskScore signature was further validated by ROC, OS, DCA, univariate, and multivariate Cox analyses.

Overall, we investigated the RNA-seq data of autophagy-related genes extracted from the ICGC and TCGA databases. Moreover, we inferred a RiskScore signature and a prognostic nomogram to effectively predict clinical outcomes of HCC patients in either ICGC or TCGA. The aforementioned results indicated that 10 hub genes are promising potential targets for treatment strategy development and the ATIC/BIRC5 autophagic signature is an applicable predictive indicator for HCC patients. However, clinical studies and experimental studies are still necessary to further confirm the clinical application and more underlying functions of the signature.

## Data Availability Statement

The datasets presented in this study can be found in online repositories. The names of the repository/repositories and accession number(s) can be found in the article/Supplementary Material.

## Author Contributions

QT, JX, and JW designed the study. SS conducted the data-mining analyses and wrote the manuscript. RW, HQ, and CL made the relative clinical statistics and pictures. All the authors have read and approved the manuscript.

## Conflict of Interest

The authors declare that the research was conducted in the absence of any commercial or financial relationships that could be construed as a potential conflict of interest.

## Publisher’s Note

All claims expressed in this article are solely those of the authors and do not necessarily represent those of their affiliated organizations, or those of the publisher, the editors and the reviewers. Any product that may be evaluated in this article, or claim that may be made by its manufacturer, is not guaranteed or endorsed by the publisher.

## Footnotes

1
http://www.autophagy.lu

2
https://icgc.org

3
https://cancergenome.nih.gov/

4
https://www.ncbi.nlm.nih.gov/geo/

5
http://www.bioconductor.org/

## Supplementary Material

Supplementary Figure 1 Quality control result of Smart-seq2 data related to HCC CSCs. **(A,B)** Gene counts of single-cell and pooled-cell samples using different classification. **(C,F)** Correlation analyses between gene counts and sequencing depth. **(D,E)** Sequencing depth of single-cell and pooled-cell samples using different classification.

Supplementary Figure 2 Characterization of single-cell RNA sequencing from 130 cells. **(A)** UMAP plot of different clusters of single-cell and pooled-cell samples. **(B)** UMAP plot of various pooled cell number of each sample. **(C)** UMAP plot of detailed information of each sample. **(D)** Top 5,000 variable genes were used for dimensional reduction analysis. **(E)** Elbow plot of 20 principal components. **(F)** Estimated *P*-value for principal components.

Supplementary Figure 3 Correlation analysis among 10 hub genes and three CSC markers in ICGC database. Only significant ones were showed.

Supplementary Figure 4 Correlation analysis among 10 hub genes and three CSC markers in TCGA database. Only significant ones were showed.

## Abbreviations

DEGs: differentially expressed genes
BP: biological process
MF: molecular function
CC: cellular component
GO: gene ontology
HADb: Human Autophagy Database
HCC: hepatocellular carcinoma
KEGG: Kyoto Encyclopedia of Genes
OS: overall survival
RNA-seq: RNA sequencing
TCGA: The Cancer Genome Atlas
ICGC: International Cancer Genome Consortium
PCA: principal component analysis
CDF: cumulative distribution function
ROC: receiver operating characteristic curve
LASSO: the least absolute shrinkage and selection operator Cox regression algorithm
DCA: decision curve analysis
UMAP: Uniform Manifold Approximation and Projection.
