# Supplementary figures and images for: Development of an Autophagy-Based and Stemness-Correlated Prognostic Model for Hepatocellular Carcinoma Using Bulk and Single-Cell RNA-Sequencing
Source: Front Cell Dev Biol. 2021 Nov 8;9:743910. doi: 10.3389/fcell.2021.743910 (PMC8606524; doi:10.3389/fcell.2021.743910)

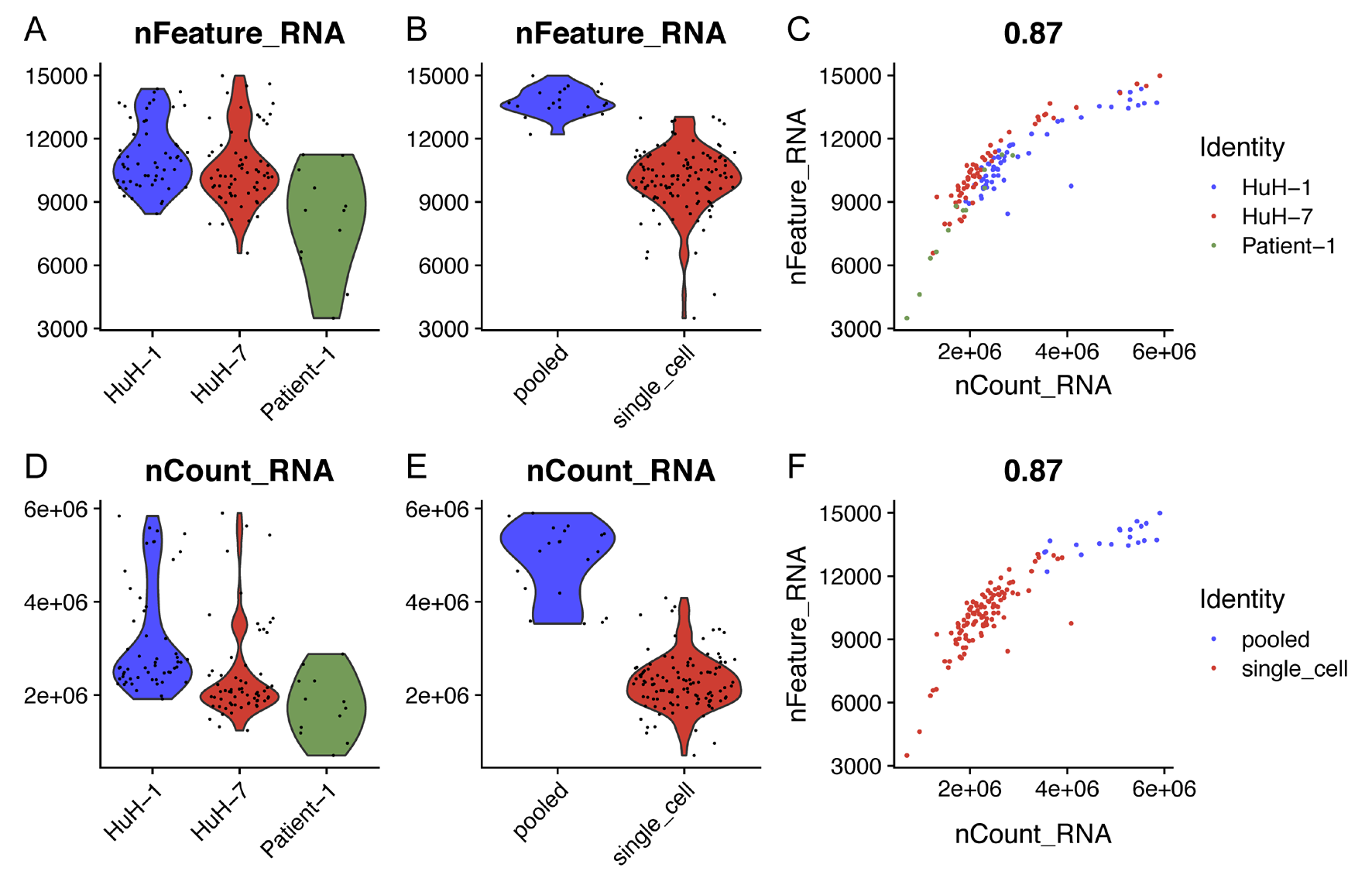

Supplement: Supplementary Figure 1 — Quality control result of Smart-seq2 data related to HCC CSCs. (A,B) Gene counts of single-cell and pooled-cell samples using different classification. (C,F) Correlation analyses between gene counts and sequencing depth. (D,E) Sequencing depth of single-cell and pooled-cell samples using different classification. [file Image_1.TIF]

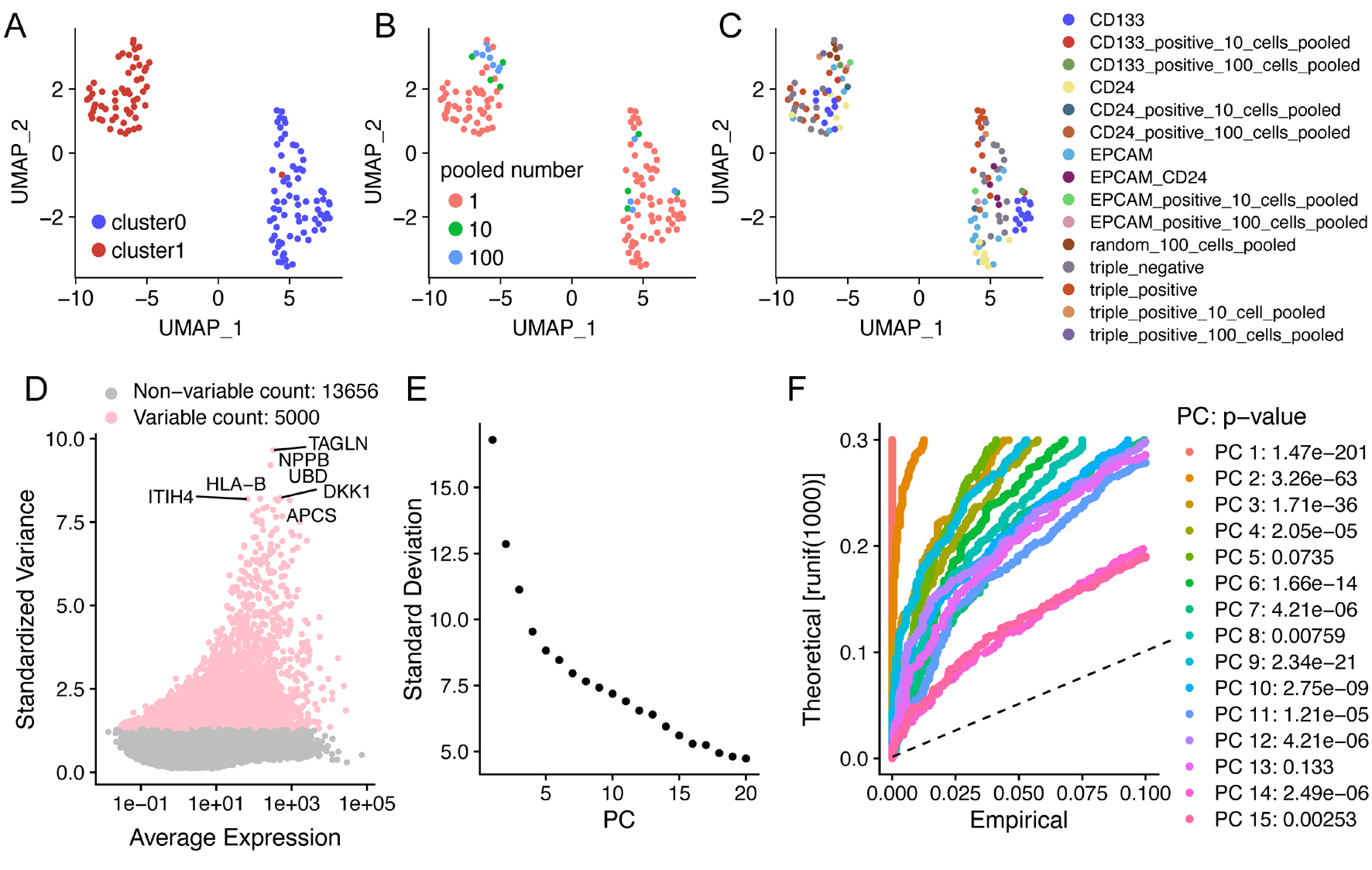

Supplement: Supplementary Figure 2 — Characterization of single-cell RNA sequencing from 130 cells. (A) UMAP plot of different clusters of single-cell and pooled-cell samples. (B) UMAP plot of various pooled cell number of each sample. (C) UMAP plot of detailed information of each sample. (D) Top 5,000 variable genes were used for dimensional reduction analysis. (E) Elbow plot of 20 principal components. (F) Estimated P-value for principal components. [file Image_2.TIF]

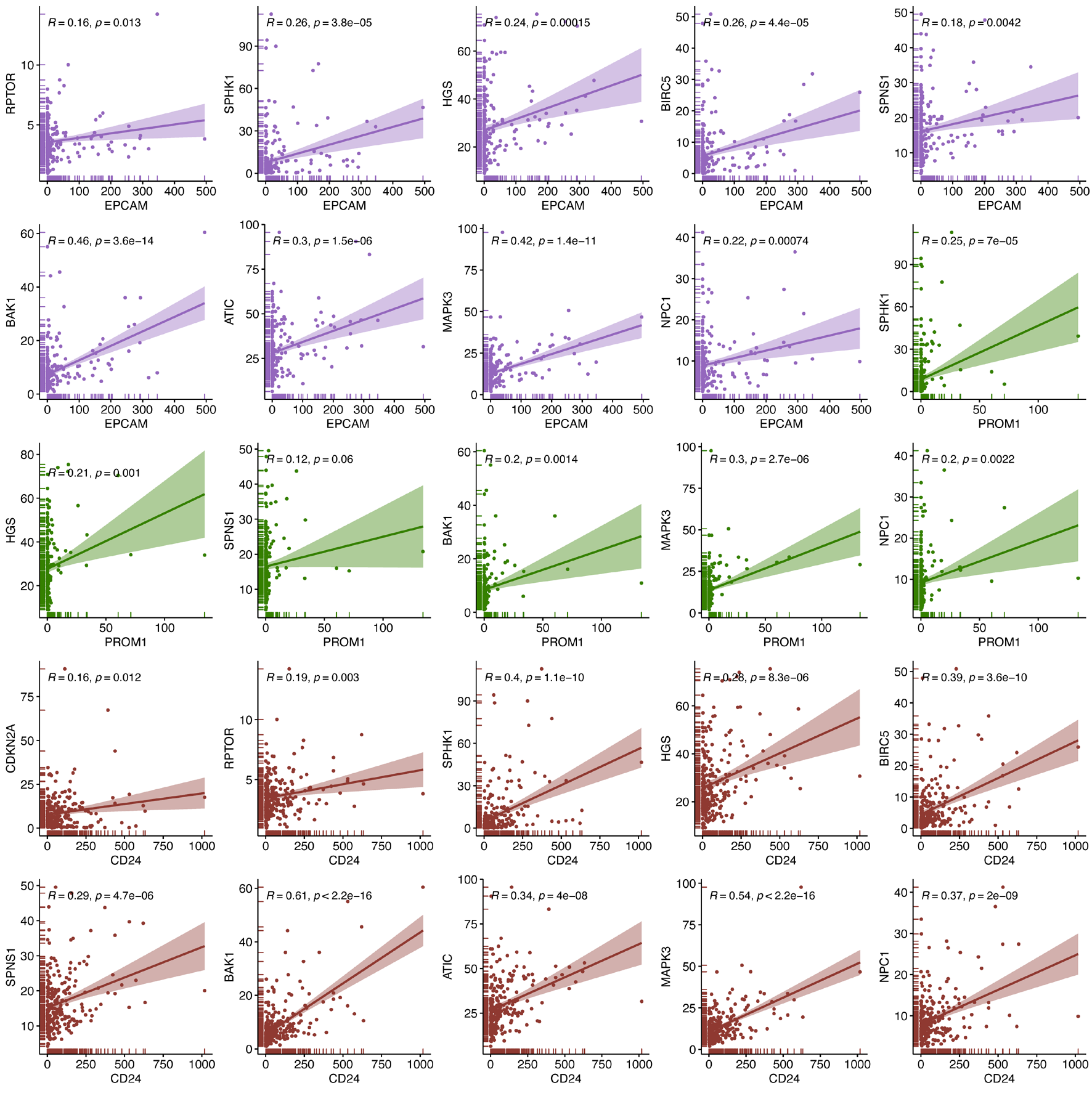

Supplement: Supplementary Figure 3 — Correlation analysis among 10 hub genes and three CSC markers in ICGC database. Only significant ones were showed. [file Image_3.TIF]

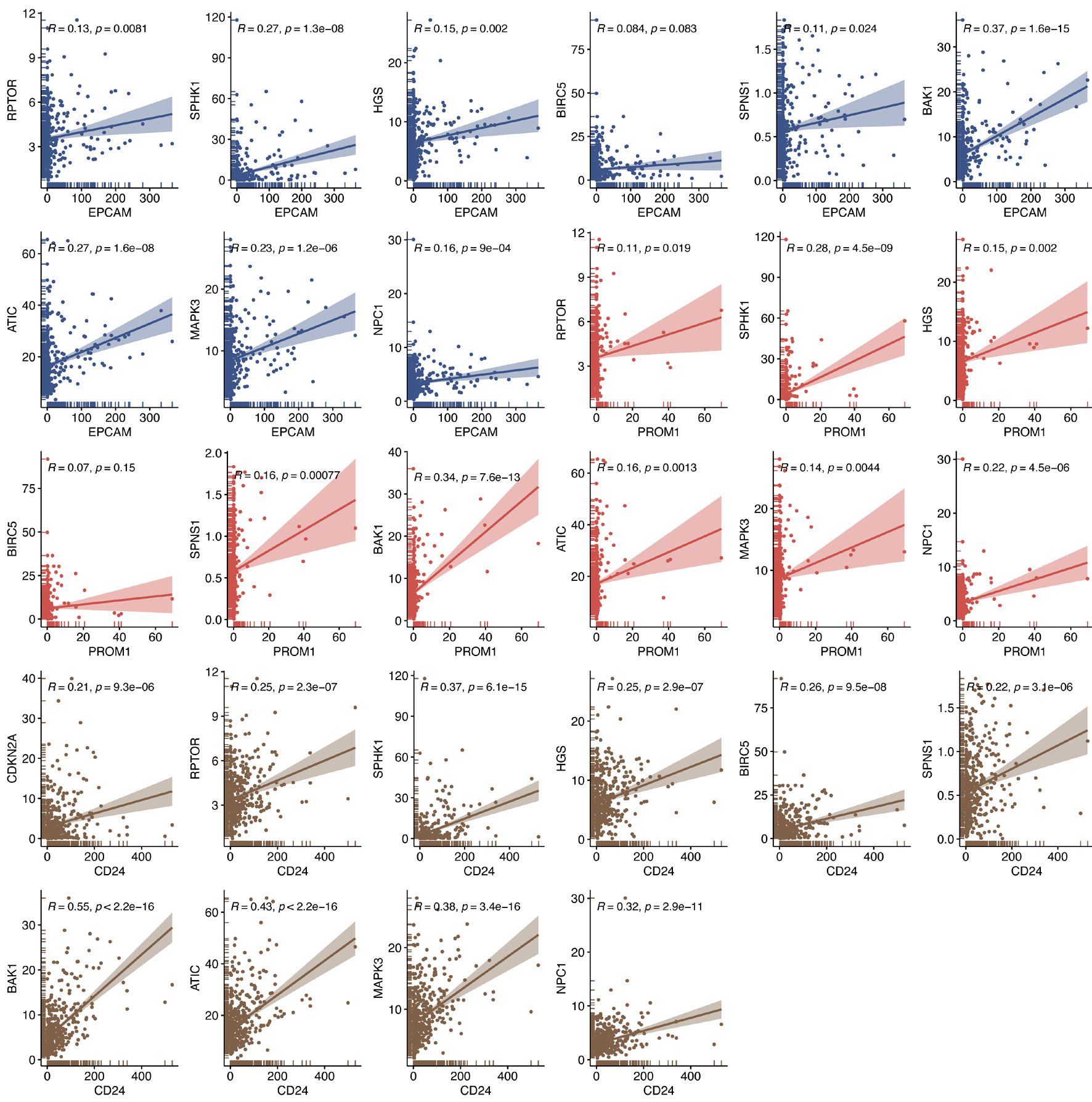

Supplement: Supplementary Figure 4 — Correlation analysis among 10 hub genes and three CSC markers in TCGA database. Only significant ones were showed. [file Image_4.TIF]
