# Supplementary material for: Development of an Autophagy-Based and Stemness-Correlated Prognostic Model for Hepatocellular Carcinoma Using Bulk and Single-Cell RNA-Sequencing
Source: Front Cell Dev Biol. 2021 Nov 8;9:743910. doi: 10.3389/fcell.2021.743910 (PMC8606524; doi:10.3389/fcell.2021.743910)
Supplement: Supplementary file 5 [file Table_1.DOCX]

Table S1 The differential analysis of autophagy-related genes in ICGC.

| gene | conMean | treatMean | logFC | pValue | fdr |
| --- | --- | --- | --- | --- | --- |
| AMBRA1 | 2.813551703 | 4.844287907 | 0.783892135 | 1.23E-47 | 2.42E-46 |
| APOL1 | 62.2328302 | 52.8760112 | -0.235062509 | 1.17E-13 | 1.93E-13 |
| ARNT | 10.23459615 | 15.66355206 | 0.613957236 | 3.21E-24 | 8.60E-24 |
| ARSA | 7.625763159 | 13.25758716 | 0.797864604 | 1.08E-28 | 3.57E-28 |
| ARSB | 1.776717976 | 2.731220762 | 0.620331235 | 4.68E-11 | 7.02E-11 |
| ATF4 | 139.1780495 | 180.4954276 | 0.375030596 | 1.56E-08 | 2.18E-08 |
| ATF6 | 12.90253352 | 20.16866919 | 0.644461513 | 3.58E-19 | 7.88E-19 |
| ATG10 | 1.083309187 | 2.124642996 | 0.971775384 | 1.34E-47 | 2.42E-46 |
| ATG12 | 5.955443911 | 8.983552146 | 0.593076959 | 4.04E-41 | 3.64E-40 |
| ATG16L1 | 6.561660955 | 8.976346288 | 0.452067282 | 5.34E-26 | 1.63E-25 |
| ATG16L2 | 9.558550527 | 10.83040319 | 0.180223184 | 0.035921269 | 0.039295089 |
| ATG2A | 7.33219809 | 5.869693671 | -0.320960549 | 8.88E-10 | 1.28E-09 |
| ATG2B | 4.714896383 | 4.622654583 | -0.028504501 | 0.205903751 | 0.218015737 |
| ATG3 | 18.13323149 | 26.40321567 | 0.542077599 | 3.10E-25 | 9.02E-25 |
| ATG4A | 5.809570903 | 7.635162392 | 0.394227232 | 2.20E-15 | 4.07E-15 |
| ATG4B | 10.74026246 | 15.69659166 | 0.54742208 | 5.33E-20 | 1.19E-19 |
| ATG4C | 2.843825409 | 4.161554729 | 0.549289714 | 5.89E-30 | 2.33E-29 |
| ATG4D | 5.253607455 | 7.791310785 | 0.568557655 | 4.70E-13 | 7.50E-13 |
| ATG5 | 6.887239505 | 8.143817967 | 0.241779466 | 4.76E-07 | 6.29E-07 |
| ATG7 | 3.976900145 | 7.788350053 | 0.96967339 | 1.78E-47 | 2.93E-46 |
| ATG9A | 7.557680239 | 13.57460847 | 0.844895202 | 1.54E-40 | 1.27E-39 |
| ATIC | 14.44391804 | 29.83487066 | 1.046537384 | 1.16E-46 | 1.53E-45 |
| BAG1 | 8.31818903 | 9.950588318 | 0.258512355 | 0.021159907 | 0.023537424 |
| BAK1 | 3.443618766 | 8.804857903 | 1.354374287 | 2.71E-32 | 1.10E-31 |
| BAX | 18.47802563 | 36.73748505 | 0.991442253 | 8.49E-35 | 3.82E-34 |
| BCL2 | 1.009866664 | 1.166996858 | 0.208635855 | 0.391870913 | 0.404116879 |
| BCL2L1 | 26.55375234 | 40.28446131 | 0.601307719 | 1.77E-16 | 3.46E-16 |
| BECN1 | 14.0127604 | 23.86314217 | 0.768042839 | 5.28E-39 | 3.37E-38 |
| BID | 9.935491224 | 16.87360542 | 0.764105067 | 1.26E-29 | 4.54E-29 |
| BIRC5 | 0.63727583 | 6.523085092 | 3.355564599 | 9.63E-61 | 1.91E-58 |
| BIRC6 | 4.170659266 | 5.126219435 | 0.297619785 | 7.58E-08 | 1.03E-07 |
| BNIP1 | 4.178554372 | 5.899804801 | 0.497663314 | 8.47E-23 | 2.10E-22 |
| BNIP3 | 61.71067932 | 69.35428266 | 0.168464796 | 0.030886862 | 0.034165356 |
| C12orf44 | 16.0750464 | 18.10875892 | 0.171864773 | 0.015058017 | 0.016940269 |
| CALCOCO2 | 24.81974371 | 30.13151105 | 0.279784806 | 1.88E-11 | 2.85E-11 |
| CAMKK2 | 7.707758088 | 9.944141805 | 0.367535577 | 8.83E-14 | 1.47E-13 |
| CANX | 76.5906471 | 153.4298012 | 1.002338597 | 4.57E-47 | 6.46E-46 |
| CAPN1 | 14.41760601 | 27.93290071 | 0.954133768 | 5.44E-36 | 2.76E-35 |
| CAPN10 | 2.702639378 | 5.006320382 | 0.889381599 | 7.92E-30 | 3.02E-29 |
| CAPN2 | 17.63970674 | 33.1950864 | 0.91214313 | 6.88E-21 | 1.58E-20 |
| CAPNS1 | 59.92048817 | 109.3632227 | 0.86800638 | 8.21E-41 | 7.07E-40 |
| CASP3 | 7.874130326 | 11.61449959 | 0.560734502 | 6.11E-24 | 1.61E-23 |
| CASP8 | 5.425957838 | 8.032445092 | 0.565961376 | 4.26E-18 | 9.08E-18 |
| CCL2 | 87.86375196 | 48.20639233 | -0.866043641 | 1.54E-16 | 3.08E-16 |
| CCR2 | 1.061378234 | 0.962700886 | -0.140779345 | 0.001373222 | 0.00161844 |
| CD46 | 44.90229045 | 76.32954882 | 0.765452625 | 4.72E-33 | 1.95E-32 |
| CDKN1A | 65.02455914 | 54.61922849 | -0.251575777 | 1.71E-06 | 2.21E-06 |
| CDKN1B | 14.14550605 | 17.61960288 | 0.316837619 | 1.73E-12 | 2.66E-12 |
| CDKN2A | 1.157308498 | 8.822295206 | 2.930380548 | 1.59E-44 | 1.66E-43 |
| CFLAR | 13.07908518 | 15.46571725 | 0.241812107 | 0.000928729 | 0.001107761 |
| CHMP2B | 9.327258931 | 13.13489353 | 0.493879433 | 5.47E-17 | 1.13E-16 |
| CHMP4B | 26.90712572 | 42.1805921 | 0.648591059 | 2.66E-24 | 7.41E-24 |
| CLN3 | 8.944156221 | 24.08887401 | 1.429349667 | 3.25E-59 | 3.22E-57 |
| CTSB | 236.6105807 | 235.5472946 | -0.006497827 | 5.13E-05 | 6.39E-05 |
| CTSD | 291.3113784 | 522.6769505 | 0.843357487 | 1.59E-18 | 3.45E-18 |
| CTSL1 | 108.1676566 | 90.95563806 | -0.250034207 | 1.65E-08 | 2.28E-08 |
| CXCR4 | 29.97708793 | 34.14991271 | 0.188021649 | 0.834907224 | 0.834907224 |
| DAPK1 | 11.30221013 | 10.21035454 | -0.146571955 | 0.000423105 | 0.000513955 |
| DAPK2 | 1.329169678 | 4.260387681 | 1.68045943 | 3.78E-39 | 2.49E-38 |
| DDIT3 | 32.88180939 | 48.03294159 | 0.546734475 | 1.10E-08 | 1.54E-08 |
| DIRAS3 | 3.263277999 | 0.887587145 | -1.878361218 | 5.68E-52 | 1.61E-50 |
| DLC1 | 9.780620782 | 6.447384755 | -0.601211956 | 5.57E-23 | 1.40E-22 |
| DNAJB1 | 135.4136393 | 124.1201712 | -0.125635467 | 0.77419615 | 0.778126079 |
| DRAM1 | 2.905219907 | 5.242400319 | 0.851580154 | 1.70E-16 | 3.37E-16 |
| EDEM1 | 14.84399649 | 12.29362978 | -0.271968621 | 2.28E-09 | 3.25E-09 |
| EEF2 | 213.6164313 | 303.3041392 | 0.505742563 | 5.90E-17 | 1.20E-16 |
| EEF2K | 2.08770623 | 3.589035641 | 0.781677531 | 3.83E-34 | 1.65E-33 |
| EIF2AK2 | 3.855898066 | 5.124678625 | 0.410394621 | 2.65E-10 | 3.95E-10 |
| EIF2AK3 | 4.220159238 | 4.723492982 | 0.162556678 | 0.001748759 | 0.002048842 |
| EIF2S1 | 8.461314669 | 10.57885156 | 0.322229274 | 2.85E-13 | 4.63E-13 |
| EIF4EBP1 | 16.81925612 | 32.62522532 | 0.955873966 | 2.22E-14 | 3.79E-14 |
| EIF4G1 | 44.87595298 | 55.28536016 | 0.300954922 | 3.44E-13 | 5.54E-13 |
| ERBB2 | 12.30690843 | 15.1838561 | 0.303069831 | 0.238387229 | 0.251067401 |
| ERN1 | 2.661758635 | 2.370405495 | -0.16724588 | 0.008224194 | 0.009358566 |
| ERO1L | 7.362767328 | 12.69404188 | 0.785831491 | 4.89E-15 | 8.97E-15 |
| FADD | 5.475228119 | 9.218397718 | 0.751596938 | 4.05E-29 | 1.38E-28 |
| FAM48A | 5.34798619 | 7.394306114 | 0.46741903 | 1.50E-16 | 3.02E-16 |
| FAS | 8.278934433 | 6.899919419 | -0.262865579 | 7.01E-09 | 9.91E-09 |
| FKBP1A | 40.81833289 | 75.6708966 | 0.890521278 | 1.63E-40 | 1.29E-39 |
| FKBP1B | 2.916910785 | 6.609003111 | 1.179991414 | 0.000429056 | 0.000518007 |
| FOS | 152.2870935 | 65.2155424 | -1.223505938 | 1.72E-28 | 5.58E-28 |
| FOXO1 | 6.713886441 | 4.754406113 | -0.497882994 | 2.25E-16 | 4.36E-16 |
| FOXO3 | 3.318687679 | 4.387505693 | 0.402788133 | 2.54E-07 | 3.42E-07 |
| GAA | 20.17599149 | 32.68471405 | 0.695976503 | 1.27E-23 | 3.26E-23 |
| GABARAP | 157.9650729 | 189.1438341 | 0.259878142 | 0.002710106 | 0.003156477 |
| GABARAPL1 | 72.51904898 | 47.14505114 | -0.621253668 | 1.02E-27 | 3.21E-27 |
| GABARAPL2 | 32.60449334 | 42.60657425 | 0.386005256 | 6.11E-15 | 1.11E-14 |
| GAPDH | 627.0732326 | 1188.504531 | 0.92244156 | 4.02E-28 | 1.28E-27 |
| GNAI3 | 8.946126758 | 11.89404499 | 0.410904333 | 8.51E-16 | 1.60E-15 |
| GNB2L1 | 300.4638384 | 518.5369395 | 0.787255398 | 5.31E-34 | 2.24E-33 |
| GOPC | 5.777559945 | 7.282197948 | 0.333913634 | 6.25E-10 | 9.17E-10 |
| HDAC1 | 17.9189271 | 32.31235942 | 0.850601841 | 2.60E-37 | 1.47E-36 |
| HDAC6 | 32.62220215 | 25.63774126 | -0.34758501 | 1.14E-12 | 1.79E-12 |
| HGS | 12.1375673 | 28.3133381 | 1.222002556 | 7.15E-53 | 2.36E-51 |
| HIF1A | 18.2969082 | 21.78048998 | 0.251436527 | 0.035725445 | 0.039295089 |
| HSP90AB1 | 156.5150944 | 367.0781237 | 1.22978534 | 1.19E-43 | 1.18E-42 |
| HSPA5 | 114.7536626 | 190.0559695 | 0.727884139 | 1.02E-25 | 3.05E-25 |
| HSPA8 | 304.4491451 | 365.8216388 | 0.264939153 | 0.000123461 | 0.000152783 |
| HSPB8 | 3.699612561 | 6.876480271 | 0.894296116 | 0.728140414 | 0.743153619 |
| IFNG | 0.81506797 | 0.642149201 | -0.344011833 | 4.20E-06 | 5.37E-06 |
| IKBKB | 6.727340663 | 9.267812111 | 0.46219248 | 4.03E-12 | 6.14E-12 |
| IKBKE | 1.038771722 | 2.416195898 | 1.217858783 | 1.62E-14 | 2.81E-14 |
| IL24 | 0.856084141 | 0.588530802 | -0.540634676 | 1.70E-12 | 2.62E-12 |
| ITGA3 | 1.891395734 | 7.768218694 | 2.038132569 | 0.020626959 | 0.023074225 |
| ITGA6 | 3.665554928 | 14.22669416 | 1.956496934 | 1.06E-55 | 5.23E-54 |
| ITGB1 | 86.95898902 | 140.6335964 | 0.693534212 | 8.78E-22 | 2.10E-21 |
| ITGB4 | 1.381699369 | 5.840093382 | 2.079547691 | 7.91E-13 | 1.25E-12 |
| ITPR1 | 1.289245227 | 1.593457223 | 0.305633586 | 0.005367091 | 0.006178395 |
| KIAA0226 | 2.032703391 | 2.926807235 | 0.525928016 | 1.81E-14 | 3.12E-14 |
| KIF5B | 10.29771785 | 16.96073363 | 0.719873927 | 3.35E-25 | 9.60E-25 |
| KLHL24 | 4.67070778 | 6.518553885 | 0.480910757 | 1.56E-14 | 2.74E-14 |
| LAMP1 | 76.54576885 | 107.1098418 | 0.48469651 | 6.48E-15 | 1.17E-14 |
| LAMP2 | 33.04850645 | 51.28447737 | 0.633937147 | 3.06E-14 | 5.18E-14 |
| MAP1LC3A | 25.30193282 | 21.97874635 | -0.203138498 | 6.15E-06 | 7.81E-06 |
| MAP1LC3B | 15.02402495 | 17.59080667 | 0.227550279 | 0.012687205 | 0.014354666 |
| MAP2K7 | 5.943417778 | 8.839701568 | 0.572704869 | 2.53E-29 | 8.80E-29 |
| MAPK1 | 8.423268729 | 15.48516026 | 0.878434215 | 1.00E-37 | 5.83E-37 |
| MAPK3 | 6.746954369 | 14.34101215 | 1.08783854 | 2.43E-35 | 1.14E-34 |
| MAPK8 | 5.433068716 | 5.73939457 | 0.079131266 | 0.409295432 | 0.41989894 |
| MAPK8IP1 | 1.693115822 | 2.305929398 | 0.445667674 | 0.072064406 | 0.077547567 |
| MAPK9 | 4.538491602 | 7.404716857 | 0.706231683 | 2.28E-35 | 1.10E-34 |
| MBTPS2 | 3.813787206 | 5.603844024 | 0.555192451 | 7.96E-16 | 1.52E-15 |
| MLST8 | 7.517087646 | 14.53008228 | 0.950797143 | 3.86E-40 | 2.83E-39 |
| MTMR14 | 7.848070751 | 13.26757305 | 0.757494539 | 2.59E-47 | 3.94E-46 |
| MTOR | 4.578025027 | 7.81631973 | 0.771764133 | 9.38E-30 | 3.51E-29 |
| MYC | 24.53742641 | 23.85971819 | -0.040406938 | 0.108551903 | 0.115555251 |
| NAMPT | 79.26784605 | 50.72184528 | -0.644128542 | 3.07E-06 | 3.95E-06 |
| NBR1 | 23.05083944 | 31.48402396 | 0.449800653 | 2.71E-16 | 5.22E-16 |
| NCKAP1 | 11.04907138 | 19.71283416 | 0.835210088 | 7.66E-30 | 2.97E-29 |
| NFE2L2 | 48.98561654 | 41.46446332 | -0.240482779 | 8.36E-08 | 1.13E-07 |
| NFKB1 | 8.055579208 | 8.126704834 | 0.012682171 | 0.367380197 | 0.380844393 |
| NLRC4 | 0.746117265 | 0.685487039 | -0.122273006 | 0.001093214 | 0.001296146 |
| NPC1 | 4.63289609 | 9.412976121 | 1.02273661 | 1.91E-36 | 1.05E-35 |
| NRG1 | 2.275242428 | 1.297918366 | -0.809820627 | 8.54E-21 | 1.94E-20 |
| P4HB | 295.3333266 | 521.4623038 | 0.8202188 | 1.95E-40 | 1.48E-39 |
| PARK2 | 0.424181794 | 0.749759017 | 0.821744267 | 0.000194639 | 0.00023937 |
| PARP1 | 14.25374915 | 28.1605096 | 0.982332002 | 6.03E-38 | 3.62E-37 |
| PEA15 | 17.38001474 | 42.08487943 | 1.275872679 | 5.21E-45 | 5.73E-44 |
| PELP1 | 6.706853929 | 9.975806193 | 0.572797256 | 5.71E-18 | 1.20E-17 |
| PEX14 | 8.72419889 | 9.081807084 | 0.057956731 | 0.732593716 | 0.743864389 |
| PEX3 | 5.333464647 | 4.944506567 | -0.109246464 | 0.000236668 | 0.000289261 |
| PIK3C3 | 4.367920864 | 5.632076206 | 0.366720137 | 1.24E-12 | 1.94E-12 |
| PIK3R4 | 5.039812646 | 5.372731451 | 0.092285626 | 0.059986712 | 0.065260269 |
| PINK1 | 31.99656669 | 25.45486447 | -0.329975724 | 1.53E-14 | 2.71E-14 |
| PPP1R15A | 35.99396157 | 34.47171576 | -0.062341789 | 0.005245831 | 0.00607412 |
| PRKAB1 | 6.098048003 | 10.8627641 | 0.832971841 | 3.97E-45 | 4.62E-44 |
| PRKAR1A | 40.60497871 | 59.66400879 | 0.555204283 | 1.67E-18 | 3.60E-18 |
| PRKCD | 4.614174541 | 8.477450532 | 0.877557883 | 1.12E-22 | 2.74E-22 |
| PRKCQ | 0.68974592 | 0.556447625 | -0.309819115 | 2.19E-05 | 2.74E-05 |
| PTEN | 9.941287012 | 10.64546986 | 0.098735086 | 0.068957089 | 0.074609309 |
| RAB11A | 10.52156511 | 15.76435368 | 0.583316698 | 2.51E-29 | 8.80E-29 |
| RAB1A | 39.06153522 | 51.97194235 | 0.411984326 | 1.70E-22 | 4.10E-22 |
| RAB24 | 11.75259596 | 22.92454347 | 0.963913543 | 2.26E-34 | 9.94E-34 |
| RAB33B | 3.967169925 | 3.287002851 | -0.271337486 | 6.46E-10 | 9.41E-10 |
| RAB5A | 14.76445156 | 17.55763704 | 0.249970929 | 6.05E-10 | 8.94E-10 |
| RAB7A | 55.89180016 | 75.2682002 | 0.429403832 | 3.17E-24 | 8.59E-24 |
| RAC1 | 48.34948227 | 86.97455928 | 0.847093022 | 4.00E-41 | 3.64E-40 |
| RAF1 | 20.70432821 | 30.63502403 | 0.565249591 | 2.26E-38 | 1.40E-37 |
| RB1 | 4.628721828 | 6.271674705 | 0.438236869 | 3.59E-08 | 4.93E-08 |
| RB1CC1 | 6.114413813 | 11.95763363 | 0.967645815 | 4.55E-35 | 2.09E-34 |
| RELA | 18.84057306 | 24.2731207 | 0.365516755 | 4.66E-23 | 1.18E-22 |
| RGS19 | 3.336192714 | 5.064314177 | 0.60216428 | 1.02E-09 | 1.46E-09 |
| RHEB | 24.34436256 | 42.97305608 | 0.819844654 | 7.21E-46 | 8.92E-45 |
| RPS6KB1 | 4.715984928 | 6.607349815 | 0.48651262 | 1.27E-24 | 3.59E-24 |
| RPTOR | 1.515831481 | 3.654099464 | 1.26940653 | 3.13E-57 | 2.07E-55 |
| SAR1A | 21.32503782 | 26.54981247 | 0.316153371 | 1.51E-15 | 2.82E-15 |
| SERPINA1 | 5582.611848 | 4120.932299 | -0.437969488 | 1.69E-13 | 2.76E-13 |
| SESN2 | 4.458590585 | 6.118065388 | 0.456487797 | 4.19E-07 | 5.57E-07 |
| SH3GLB1 | 7.159146867 | 8.891617551 | 0.31265822 | 1.12E-06 | 1.46E-06 |
| SIRT1 | 4.709162059 | 5.081785396 | 0.10986508 | 0.315233113 | 0.329995386 |
| SIRT2 | 15.95958781 | 20.62171277 | 0.369740772 | 7.12E-18 | 1.48E-17 |
| SPHK1 | 3.214621558 | 9.473935765 | 1.559314984 | 0.0056943 | 0.006517176 |
| SPNS1 | 7.418350095 | 16.70532224 | 1.171137552 | 3.33E-53 | 1.32E-51 |
| SQSTM1 | 129.2870159 | 331.00785 | 1.356288037 | 3.03E-39 | 2.07E-38 |
| ST13 | 53.6977055 | 63.80248731 | 0.248752225 | 6.31E-07 | 8.27E-07 |
| STK11 | 7.284089641 | 11.58162771 | 0.669017446 | 2.19E-35 | 1.09E-34 |
| TBK1 | 6.818446799 | 9.027568863 | 0.404894381 | 2.93E-24 | 8.07E-24 |
| TM9SF1 | 12.60555122 | 19.33022675 | 0.616799355 | 2.30E-36 | 1.23E-35 |
| TMEM74 | 0.095418941 | 0.847425082 | 3.150738249 | 2.47E-36 | 1.29E-35 |
| TNFSF10 | 48.34566779 | 45.02252192 | -0.102739746 | 0.000638001 | 0.000765602 |
| TP53 | 5.897628446 | 9.177953207 | 0.638037517 | 1.29E-14 | 2.30E-14 |
| TP53INP2 | 11.17590462 | 13.92701984 | 0.317494964 | 0.752181142 | 0.75985646 |
| TP73 | 0.191177347 | 0.757121213 | 1.985612706 | 1.46E-39 | 1.03E-38 |
| TSC1 | 3.378869086 | 5.41444511 | 0.680273039 | 7.67E-24 | 2.00E-23 |
| TSC2 | 7.086074267 | 11.30332447 | 0.673688661 | 1.11E-25 | 3.27E-25 |
| TUSC1 | 2.843773565 | 2.436240518 | -0.223150025 | 1.51E-05 | 1.90E-05 |
| ULK1 | 6.74120483 | 11.33731156 | 0.750000205 | 4.82E-26 | 1.49E-25 |
| ULK3 | 10.14631071 | 18.67887985 | 0.880452695 | 1.13E-29 | 4.14E-29 |
| USP10 | 10.18684212 | 12.30963482 | 0.273081073 | 2.65E-07 | 3.55E-07 |
| UVRAG | 2.911374643 | 3.905903289 | 0.423955727 | 1.07E-21 | 2.51E-21 |
| VAMP3 | 16.14220083 | 22.5013815 | 0.479176291 | 8.83E-14 | 1.47E-13 |
| VEGFA | 58.20151762 | 67.7123045 | 0.218361248 | 0.316662239 | 0.329995386 |
| WDFY3 | 1.731105945 | 1.968347578 | 0.185290978 | 0.100028477 | 0.107057505 |
| WDR45 | 19.55800384 | 29.79876577 | 0.607493446 | 6.46E-29 | 2.17E-28 |
| WDR45L | 14.14318921 | 26.61743874 | 0.912264278 | 7.33E-48 | 1.61E-46 |
| WIPI1 | 6.447227598 | 11.96990899 | 0.892661364 | 4.46E-20 | 1.00E-19 |
| WIPI2 | 7.576673792 | 13.96430346 | 0.882107073 | 2.32E-51 | 5.73E-50 |
| ZFYVE1 | 3.517574914 | 4.951263301 | 0.493215523 | 2.18E-21 | 5.07E-21 |
