# Supplementary material for: Development of an Autophagy-Based and Stemness-Correlated Prognostic Model for Hepatocellular Carcinoma Using Bulk and Single-Cell RNA-Sequencing
Source: Front Cell Dev Biol. 2021 Nov 8;9:743910. doi: 10.3389/fcell.2021.743910 (PMC8606524; doi:10.3389/fcell.2021.743910)
Supplement: Supplementary file 6 [file Table_2.DOCX]

Table S2 The differential analysis of autophagy-related genes in TCGA.

| gene | conMean | treatMean | logFC | pValue | fdr |
| --- | --- | --- | --- | --- | --- |
| AMBRA1 | 1.920238008 | 3.655057902 | 0.928609121 | 3.52E-19 | 1.61E-18 |
| APOL1 | 75.9029008 | 58.2519145 | -0.381849557 | 3.78E-06 | 5.65E-06 |
| ARNT | 6.59305832 | 10.73648202 | 0.703501601 | 1.09E-09 | 2.06E-09 |
| ARSA | 13.84392328 | 23.18197984 | 0.743750933 | 1.06E-09 | 2.02E-09 |
| ARSB | 1.361204242 | 2.312439374 | 0.76453199 | 1.93E-06 | 2.95E-06 |
| ATF4 | 66.095225 | 102.7828398 | 0.636981464 | 4.89E-09 | 8.97E-09 |
| ATF6 | 6.04478824 | 10.50125927 | 0.796798635 | 6.83E-13 | 1.77E-12 |
| ATG10 | 0.643589542 | 1.307517436 | 1.022617397 | 1.11E-22 | 9.03E-22 |
| ATG12 | 2.153676312 | 3.803706198 | 0.82060438 | 1.68E-22 | 1.31E-21 |
| ATG16L1 | 2.51868182 | 3.699242391 | 0.554560955 | 3.39E-12 | 8.13E-12 |
| ATG16L2 | 0.738395354 | 1.502660121 | 1.02505335 | 2.00E-13 | 5.42E-13 |
| ATG2A | 6.86254102 | 6.165903575 | -0.15443054 | 0.039042203 | 0.043717916 |
| ATG2B | 1.705549946 | 1.918096968 | 0.169438652 | 0.460584661 | 0.473238085 |
| ATG3 | 3.36897934 | 5.589312663 | 0.7303593 | 5.80E-19 | 2.52E-18 |
| ATG4A | 4.14087232 | 5.870545556 | 0.503559862 | 5.97E-09 | 1.08E-08 |
| ATG4B | 3.06116016 | 6.377106909 | 1.058823539 | 4.52E-24 | 7.04E-23 |
| ATG4C | 2.13154142 | 3.044659264 | 0.514383691 | 3.97E-09 | 7.36E-09 |
| ATG4D | 2.45024504 | 4.63756196 | 0.920440522 | 2.56E-12 | 6.31E-12 |
| ATG5 | 4.75831536 | 5.666518369 | 0.252011694 | 0.001653503 | 0.002047715 |
| ATG7 | 1.02359306 | 1.851176843 | 0.854800451 | 1.89E-22 | 1.42E-21 |
| ATG9A | 6.23370218 | 10.29821287 | 0.724232862 | 3.76E-17 | 1.30E-16 |
| ATIC | 7.75802162 | 18.69461756 | 1.268862257 | 1.56E-23 | 2.13E-22 |
| BAG1 | 5.2707849 | 7.050046316 | 0.419614918 | 5.05E-05 | 7.05E-05 |
| BAG3 | 9.00166868 | 13.63911831 | 0.599486016 | 5.90E-06 | 8.55E-06 |
| BAK1 | 2.43296064 | 7.457171263 | 1.615915494 | 1.55E-17 | 5.91E-17 |
| BAX | 7.3216181 | 21.10099463 | 1.527076575 | 7.52E-23 | 6.39E-22 |
| BCL2 | 0.525099866 | 0.749869446 | 0.514047614 | 0.159987418 | 0.170957983 |
| BCL2L1 | 20.41549924 | 34.11325964 | 0.740667768 | 2.90E-08 | 4.98E-08 |
| BECN1 | 3.10369122 | 5.087718246 | 0.713033744 | 4.76E-11 | 1.02E-10 |
| BID | 5.5365728 | 10.65509055 | 0.944477741 | 9.98E-15 | 2.92E-14 |
| BIRC5 | 0.250505105 | 7.049579483 | 4.814625294 | 2.35E-28 | 4.40E-26 |
| BIRC6 | 2.22498909 | 2.955056169 | 0.409387291 | 0.001254584 | 0.001574545 |
| BNIP1 | 1.86454484 | 3.503518699 | 0.909981104 | 4.57E-18 | 1.90E-17 |
| BNIP3 | 22.5036146 | 25.23572214 | 0.165310621 | 0.252370007 | 0.2666282 |
| CALCOCO2 | 9.08517126 | 11.71289998 | 0.366512699 | 1.12E-05 | 1.61E-05 |
| CAMKK2 | 4.4827044 | 7.135552898 | 0.670655853 | 3.70E-12 | 8.76E-12 |
| CANX | 60.5451652 | 129.9874271 | 1.102288423 | 2.00E-21 | 1.29E-20 |
| CAPN1 | 9.0649984 | 17.4544332 | 0.945214838 | 4.40E-17 | 1.49E-16 |
| CAPN10 | 0.57984911 | 1.6393835 | 1.499403951 | 4.96E-26 | 1.53E-24 |
| CAPN2 | 4.52056264 | 12.08959613 | 1.4191918 | 3.60E-17 | 1.27E-16 |
| CAPNS1 | 31.432903 | 67.47791914 | 1.102139966 | 5.81E-23 | 5.44E-22 |
| CASP1 | 2.356665298 | 2.667478406 | 0.178729715 | 0.697342752 | 0.708712471 |
| CASP3 | 4.1860116 | 8.44081184 | 1.011805455 | 1.98E-17 | 7.25E-17 |
| CASP4 | 4.20085128 | 5.10681863 | 0.28174311 | 0.032860592 | 0.037242004 |
| CASP8 | 1.376885276 | 2.831424064 | 1.040119482 | 1.52E-15 | 4.73E-15 |
| CCL2 | 12.78402676 | 7.827972283 | -0.70763178 | 5.47E-07 | 8.81E-07 |
| CD46 | 11.0897999 | 24.19238825 | 1.125319864 | 1.83E-17 | 6.86E-17 |
| CDKN1A | 50.49138318 | 48.30619768 | -0.063828901 | 0.572328179 | 0.584838084 |
| CDKN1B | 11.14478078 | 15.33984654 | 0.460915812 | 6.79E-07 | 1.08E-06 |
| CDKN2A | 0.162719125 | 4.097589759 | 4.654319821 | 1.87E-25 | 4.37E-24 |
| CFLAR | 2.67542836 | 3.430899529 | 0.35881698 | 0.000109914 | 0.000146813 |
| CHMP2B | 7.37121326 | 9.876101316 | 0.422039538 | 1.91E-05 | 2.70E-05 |
| CHMP4B | 32.0528566 | 59.56277227 | 0.893957966 | 6.59E-18 | 2.62E-17 |
| CLN3 | 2.44659002 | 7.973021551 | 1.704354202 | 7.73E-27 | 3.91E-25 |
| CTSB | 152.5673934 | 135.9559648 | -0.166307215 | 0.000889768 | 0.001131881 |
| CTSD | 272.813656 | 482.4807219 | 0.822555437 | 6.30E-10 | 1.22E-09 |
| CXCR4 | 10.60366533 | 12.76883288 | 0.268063621 | 0.167799371 | 0.178286832 |
| DAPK1 | 5.52550798 | 5.522408116 | -0.000809593 | 0.014400199 | 0.016622452 |
| DAPK2 | 0.207441254 | 0.926243266 | 2.158688316 | 1.19E-20 | 7.20E-20 |
| DDIT3 | 9.15750984 | 26.0192576 | 1.506552547 | 3.18E-17 | 1.14E-16 |
| DIRAS3 | 2.884783714 | 0.621647671 | -2.214294111 | 2.04E-22 | 1.46E-21 |
| DLC1 | 4.38615618 | 2.795294495 | -0.649956899 | 1.55E-10 | 3.30E-10 |
| DNAJB1 | 21.3038263 | 32.89537578 | 0.626772222 | 1.52E-06 | 2.35E-06 |
| DRAM1 | 1.68390873 | 3.868698368 | 1.200034306 | 1.11E-11 | 2.54E-11 |
| EDEM1 | 11.2639317 | 10.17417894 | -0.146798119 | 0.015994985 | 0.018350075 |
| EEF2 | 268.409438 | 389.7671708 | 0.538177179 | 1.05E-08 | 1.88E-08 |
| EEF2K | 1.693265162 | 3.160030778 | 0.900130696 | 1.50E-15 | 4.73E-15 |
| EIF2AK2 | 2.386024176 | 3.420289268 | 0.519509684 | 8.90E-07 | 1.40E-06 |
| EIF2AK3 | 1.950976156 | 2.560898309 | 0.39245382 | 0.000889768 | 0.001131881 |
| EIF2S1 | 5.73759198 | 7.850115623 | 0.452268527 | 2.30E-08 | 4.02E-08 |
| EIF4EBP1 | 19.2051005 | 34.86899661 | 0.860455336 | 1.76E-05 | 2.52E-05 |
| EIF4G1 | 34.9092922 | 47.35035816 | 0.439764232 | 2.80E-09 | 5.23E-09 |
| ERBB2 | 6.04569194 | 9.435531437 | 0.642196309 | 0.027450452 | 0.031300211 |
| ERN1 | 3.0006375 | 3.689129615 | 0.298011437 | 0.122894449 | 0.133611988 |
| FADD | 2.29921568 | 4.460190728 | 0.955963599 | 1.97E-15 | 5.93E-15 |
| FAS | 3.60335702 | 3.464695826 | -0.056612901 | 0.041556198 | 0.046256006 |
| FKBP1A | 22.1602108 | 49.66569179 | 1.164278002 | 2.89E-23 | 3.37E-22 |
| FKBP1B | 1.610316392 | 3.816892546 | 1.245054398 | 0.333824964 | 0.346807046 |
| FOS | 113.2004605 | 21.16320425 | -2.419249845 | 6.68E-21 | 4.16E-20 |
| FOXO1 | 9.47903652 | 4.497195139 | -1.075714941 | 4.28E-15 | 1.27E-14 |
| FOXO3 | 3.5122129 | 4.527969937 | 0.36648408 | 0.035460273 | 0.039946211 |
| GAA | 20.41744244 | 34.96541419 | 0.776126435 | 1.55E-11 | 3.46E-11 |
| GABARAP | 16.29925 | 19.10437768 | 0.229097682 | 0.005475103 | 0.006521302 |
| GABARAPL1 | 29.2948442 | 20.99723476 | -0.480447433 | 8.06E-09 | 1.45E-08 |
| GABARAPL2 | 11.33231702 | 16.68803921 | 0.558371586 | 5.07E-13 | 1.35E-12 |
| GAPDH | 278.514832 | 661.6961246 | 1.248414671 | 5.02E-20 | 2.61E-19 |
| GNAI3 | 1.49649748 | 2.230661646 | 0.575881848 | 2.26E-10 | 4.60E-10 |
| GOPC | 3.25950112 | 4.318539803 | 0.405892416 | 0.000281594 | 0.000368238 |
| HDAC1 | 9.67253196 | 19.69687818 | 1.026001495 | 6.77E-19 | 2.88E-18 |
| HDAC6 | 8.2466034 | 7.594234246 | -0.118895526 | 0.009955437 | 0.011708596 |
| HGS | 2.40829612 | 7.270745895 | 1.594090581 | 3.22E-22 | 2.23E-21 |
| HIF1A | 9.27623998 | 13.76263978 | 0.569145168 | 0.148616665 | 0.160643448 |
| HSP90AB1 | 114.1781854 | 354.4065151 | 1.634118086 | 3.53E-26 | 1.32E-24 |
| HSPA5 | 110.1952336 | 231.5746734 | 1.071415656 | 1.04E-19 | 5.10E-19 |
| HSPA8 | 97.4611932 | 145.4438153 | 0.57756216 | 5.94E-07 | 9.49E-07 |
| HSPB8 | 1.74722711 | 7.09746478 | 2.022236638 | 0.009572811 | 0.011329846 |
| IKBKB | 1.318682464 | 2.238642509 | 0.763526953 | 1.74E-10 | 3.65E-10 |
| IKBKE | 0.548463181 | 2.00315805 | 1.868809577 | 1.30E-11 | 2.93E-11 |
| ITGA3 | 0.642552266 | 2.748765214 | 2.096897969 | 0.000448073 | 0.000581873 |
| ITGA6 | 2.511066176 | 10.4672062 | 2.059504469 | 1.59E-23 | 2.13E-22 |
| ITGB1 | 20.42827626 | 34.85764823 | 0.770907763 | 6.33E-05 | 8.76E-05 |
| ITGB4 | 0.692499462 | 3.586313499 | 2.372616752 | 1.85E-10 | 3.85E-10 |
| ITPR1 | 0.520471766 | 0.934722194 | 0.844717745 | 0.001688578 | 0.002077396 |
| KIF5B | 7.16456502 | 13.38184532 | 0.901326051 | 1.39E-12 | 3.48E-12 |
| KLHL24 | 3.39553536 | 4.318505482 | 0.346893065 | 0.004792311 | 0.005781692 |
| LAMP1 | 51.8831712 | 71.08868872 | 0.454353361 | 2.72E-06 | 4.14E-06 |
| LAMP2 | 37.7636446 | 56.20367623 | 0.573666492 | 2.89E-06 | 4.36E-06 |
| MAP1LC3B | 6.9932202 | 8.887481457 | 0.345817712 | 0.012725566 | 0.014873005 |
| MAP2K7 | 4.06799772 | 6.771536567 | 0.73516437 | 1.66E-19 | 7.78E-19 |
| MAPK1 | 4.49492406 | 7.752070525 | 0.786284954 | 7.34E-13 | 1.88E-12 |
| MAPK3 | 3.65701124 | 9.508187623 | 1.378505311 | 5.73E-26 | 1.53E-24 |
| MAPK8 | 1.93706992 | 2.331422452 | 0.267334414 | 0.013774286 | 0.015998705 |
| MAPK8IP1 | 1.516339596 | 2.149050855 | 0.50310673 | 0.313350383 | 0.327354869 |
| MAPK9 | 1.89664236 | 3.660024985 | 0.948405834 | 1.34E-19 | 6.44E-19 |
| MBTPS2 | 2.208361 | 2.960074095 | 0.422657261 | 0.000191501 | 0.000252188 |
| MLST8 | 4.34760994 | 8.973866559 | 1.045507221 | 4.72E-23 | 4.90E-22 |
| MTMR14 | 3.94736996 | 7.201176666 | 0.867340921 | 5.74E-23 | 5.44E-22 |
| MTOR | 2.937850666 | 5.183880968 | 0.819271527 | 3.61E-10 | 7.19E-10 |
| MYC | 25.68397402 | 17.36398897 | -0.564770034 | 0.000101384 | 0.000137382 |
| NAF1 | 0.831825578 | 0.961360946 | 0.208797149 | 0.048237584 | 0.053375314 |
| NAMPT | 30.29449552 | 16.41230958 | -0.884277408 | 2.28E-07 | 3.77E-07 |
| NBR1 | 13.67961408 | 17.32169874 | 0.340552892 | 0.005152759 | 0.006176704 |
| NCKAP1 | 2.108860668 | 3.670997808 | 0.799708473 | 2.19E-10 | 4.50E-10 |
| NFE2L2 | 15.58383872 | 13.16981111 | -0.242815998 | 0.001994804 | 0.002438094 |
| NFKB1 | 4.58957094 | 5.149335951 | 0.166027109 | 0.312172298 | 0.327354869 |
| NPC1 | 1.411265224 | 3.746963768 | 1.408732883 | 1.77E-20 | 9.73E-20 |
| NRG1 | 1.11742052 | 0.460111121 | -1.280117987 | 5.44E-12 | 1.26E-11 |
| P4HB | 222.7975258 | 397.6727754 | 0.835848587 | 1.36E-17 | 5.31E-17 |
| PARP1 | 7.88800852 | 20.10034408 | 1.349487183 | 6.82E-23 | 6.07E-22 |
| PEA15 | 12.16323068 | 43.36823527 | 1.834112264 | 3.57E-27 | 3.34E-25 |
| PELP1 | 3.78819386 | 7.723468171 | 1.027738663 | 5.19E-20 | 2.62E-19 |
| PEX14 | 7.81511922 | 8.262929254 | 0.080385434 | 0.881319133 | 0.881319133 |
| PEX3 | 6.59959306 | 5.506433765 | -0.261258807 | 3.27E-05 | 4.60E-05 |
| PIK3C3 | 0.909520322 | 1.266871369 | 0.47809227 | 2.98E-07 | 4.89E-07 |
| PIK3R4 | 4.41955142 | 4.645649979 | 0.071980515 | 0.813966558 | 0.818342722 |
| PINK1 | 9.085302 | 6.857324699 | -0.405888635 | 1.44E-06 | 2.24E-06 |
| PPP1R15A | 15.5574138 | 12.96851749 | -0.262588687 | 6.77E-05 | 9.30E-05 |
| PRKAB1 | 2.65650378 | 5.086577391 | 0.93716647 | 1.52E-20 | 8.89E-20 |
| PRKAR1A | 18.7262204 | 27.63748759 | 0.561566729 | 3.84E-06 | 5.71E-06 |
| PRKCD | 2.370244438 | 6.311840997 | 1.413025014 | 8.94E-16 | 2.88E-15 |
| PTEN | 5.55217968 | 6.026167145 | 0.118186432 | 0.435589203 | 0.450028624 |
| RAB11A | 8.44435498 | 13.85839696 | 0.714701254 | 5.02E-19 | 2.24E-18 |
| RAB1A | 30.5916808 | 47.19989571 | 0.625644297 | 2.09E-14 | 5.93E-14 |
| RAB24 | 1.364915904 | 4.436358589 | 1.700563917 | 8.37E-27 | 3.91E-25 |
| RAB33B | 2.341522052 | 2.00683333 | -0.222525821 | 0.000502725 | 0.000648342 |
| RAB5A | 7.72042656 | 9.975231356 | 0.369669742 | 3.37E-07 | 5.49E-07 |
| RAB7A | 54.02592 | 69.90600388 | 0.371764632 | 2.17E-08 | 3.83E-08 |
| RAC1 | 34.804967 | 64.0561561 | 0.88004402 | 1.71E-20 | 9.68E-20 |
| RAF1 | 9.16808486 | 14.02189836 | 0.61298938 | 7.54E-13 | 1.90E-12 |
| RB1 | 2.418638778 | 3.221056036 | 0.413338439 | 0.069442876 | 0.075940455 |
| RB1CC1 | 3.71849764 | 7.510565956 | 1.014201769 | 4.73E-14 | 1.30E-13 |
| RELA | 8.40284048 | 12.90864967 | 0.619389092 | 7.56E-17 | 2.53E-16 |
| RGS19 | 2.01903901 | 4.375760448 | 1.115864974 | 5.15E-10 | 1.01E-09 |
| RHEB | 6.86592528 | 15.94083991 | 1.215201584 | 2.98E-25 | 6.18E-24 |
| RPS6KB1 | 1.81607934 | 3.019498793 | 0.733481864 | 4.33E-12 | 1.01E-11 |
| RPTOR | 1.577082004 | 3.888593138 | 1.301990616 | 4.43E-25 | 8.29E-24 |
| SAR1A | 9.17006332 | 12.57976479 | 0.456101346 | 3.39E-12 | 8.13E-12 |
| SERPINA1 | 5515.47242 | 3838.021099 | -0.523121821 | 5.35E-10 | 1.04E-09 |
| SESN2 | 5.26139074 | 8.040982473 | 0.611927589 | 4.62E-06 | 6.80E-06 |
| SH3GLB1 | 5.88232056 | 7.652550765 | 0.379555303 | 0.001396123 | 0.0017405 |
| SIRT1 | 3.35522178 | 3.212805999 | -0.062574264 | 0.066842656 | 0.073526922 |
| SIRT2 | 5.52081948 | 8.40128166 | 0.605727007 | 1.19E-14 | 3.42E-14 |
| SPHK1 | 0.741709572 | 5.347719945 | 2.849997622 | 8.75E-05 | 0.000119424 |
| SPNS1 | 0.255314144 | 0.635611344 | 1.315871408 | 2.15E-14 | 6.01E-14 |
| SQSTM1 | 29.986829 | 99.91756718 | 1.73640938 | 9.02E-17 | 2.96E-16 |
| ST13 | 46.1105224 | 54.4539042 | 0.239939479 | 0.001227917 | 0.001551489 |
| STK11 | 3.37051652 | 5.995016242 | 0.830793966 | 3.56E-20 | 1.90E-19 |
| TBK1 | 3.241287942 | 4.590454682 | 0.50206987 | 2.65E-10 | 5.34E-10 |
| TM9SF1 | 1.04022104 | 1.732934484 | 0.736326989 | 5.75E-13 | 1.52E-12 |
| TMEM74 | 0.124971538 | 0.630006118 | 2.333766274 | 3.59E-11 | 7.81E-11 |
| TP53 | 4.95454942 | 8.223166971 | 0.730940262 | 5.80E-06 | 8.47E-06 |
| TP53INP2 | 12.59505756 | 15.80121315 | 0.327177611 | 0.157806739 | 0.169596898 |
| TSC1 | 0.723671554 | 2.144580065 | 1.567288209 | 2.41E-24 | 4.09E-23 |
| TSC2 | 2.49592088 | 5.605778719 | 1.167342595 | 4.51E-22 | 3.01E-21 |
| TUSC1 | 4.08852002 | 3.602008781 | -0.182777007 | 0.002710042 | 0.003290766 |
| ULK1 | 4.46900744 | 8.45461877 | 0.919785257 | 1.58E-15 | 4.84E-15 |
| ULK2 | 0.504909922 | 0.706007799 | 0.483658092 | 0.742382776 | 0.750408536 |
| ULK3 | 3.68135108 | 8.785389612 | 1.254870926 | 3.47E-23 | 3.82E-22 |
| USP10 | 6.70473044 | 8.552398944 | 0.351149822 | 0.00012214 | 0.000161987 |
| UVRAG | 1.736166088 | 2.472923162 | 0.510312446 | 2.59E-08 | 4.49E-08 |
| VAMP3 | 12.28937288 | 17.87864391 | 0.540826015 | 6.95E-08 | 1.18E-07 |
| VAMP7 | 10.89329158 | 18.78678583 | 0.786278309 | 3.25E-11 | 7.15E-11 |
| VEGFA | 5.89769448 | 10.54616655 | 0.838495693 | 1.07E-07 | 1.81E-07 |
| WDR45 | 6.12362316 | 10.01854802 | 0.710216026 | 6.59E-18 | 2.62E-17 |
| WIPI1 | 2.61044624 | 5.01231442 | 0.941180469 | 1.77E-07 | 2.96E-07 |
| WIPI2 | 4.7726463 | 8.428914067 | 0.820557352 | 1.76E-23 | 2.19E-22 |
| ZFYVE1 | 2.95946314 | 3.786586866 | 0.355562537 | 0.000103456 | 0.000139181 |
